# Biomolecular dynamics with machine-learned quantum-mechanical force fields trained on diverse chemical fragments

**DOI:** 10.1126/sciadv.adn4397

**Published:** 2024-04-05

**Authors:** Oliver T. Unke, Martin Stöhr, Stefan Ganscha, Thomas Unterthiner, Hartmut Maennel, Sergii Kashubin, Daniel Ahlin, Michael Gastegger, Leonardo Medrano Sandonas, Joshua T. Berryman, Alexandre Tkatchenko, Klaus-Robert Müller

**Affiliations:** ^1^ Google DeepMind, Tucholskystraße 2, 10117 Berlin, Germany and Brandschenkestrasse 110, 8002 Zürich, Switzerland.; ^2^ Machine Learning Group, Technische Universität Berlin, 10587 Berlin, Germany.; ^3^ DFG Cluster of Excellence “Unifying Systems in Catalysis” (UniSysCat), Technische Universität Berlin, 10623 Berlin, Germany.; ^4^ Department of Physics and Materials Science, University of Luxembourg, L-1511 Luxembourg City, Luxembourg.; ^5^ BASLEARN — TU Berlin/BASF Joint Lab for Machine Learning, Technische Universität Berlin, 10587 Berlin, Germany.; ^6^ Department of Artificial Intelligence, Korea University, Anam-dong, Seongbuk-gu, Seoul 02841, Korea.; ^7^ Max Planck Institute for Informatics, Stuhlsatzenhausweg, 66123 Saarbrücken, Germany.; ^8^ BIFOLD — Berlin Institute for the Foundations of Learning and Data, Berlin, Germany.

## Abstract

Molecular dynamics (MD) simulations allow insights into complex processes, but accurate MD simulations require costly quantum-mechanical calculations. For larger systems, efficient but less reliable empirical force fields are used. Machine-learned force fields (MLFFs) offer similar accuracy as ab initio methods at orders-of-magnitude speedup, but struggle to model long-range interactions in large molecules. This work proposes a general approach to constructing accurate MLFFs for large-scale molecular simulations (GEMS) by training on “bottom-up” and “top-down” molecular fragments, from which the relevant interactions can be learned. GEMS allows nanosecond-scale MD simulations of >25,000 atoms at essentially ab initio quality, correctly predicts dynamical oscillations between different helical motifs in polyalanine, and yields good agreement with terahertz vibrational spectroscopy for large-scale protein-water fluctuations in solvated crambin. Our analyses indicate that simulations at ab initio accuracy might be necessary to understand dynamic biomolecular processes.

## Teaser

The GEMS method enables molecular dynamics simulations of large heterogeneous systems at ab initio quality.

## INTRODUCTION

Molecular dynamics (MD) simulations allow to determine the motion of individual atoms in chemical and biological processes, enabling mechanistic insights into molecular properties and functions, as well as providing a detailed interpretation of experimental studies. MD simulations require a reliable model of the forces acting on each atom at every time step of the dynamics (*1*). It is most desirable to obtain atomic forces from accurate solutions to the many-body Schrödinger equation, but this is only feasible for short MD simulations of few atoms for the foreseeable future (*2*). We remark that while there is always a unique exact solution to the Schrödinger equation for every atomic configuration, the proliferation of approximate empirical force fields (FFs) reflects the grand challenge of accurately capturing [and even fundamentally understanding (*3*)] interatomic interactions at all relevant length and timescales.

For larger systems, it is common practice to derive the forces from empirical models of the potential energy. Such force fields (FFs) approximate the interactions between atoms with computationally efficient, albeit rather rigid, terms and enable MD simulations of proteins at millisecond timescales (*4*).

A disadvantage of FFs is their limited accuracy due to the neglect of important quantum-mechanical effects, such as changes to hybridization states, interactions between orbitals delocalized over several atoms, or electronic correlations between distant molecular fragments. Further, many FFs require a predetermined covalent bonding structure, preventing bond breaking and formation. When additional accuracy and flexibility is required, for example, to study an enzymatic reaction, a possible alternative is quantum mechanics/molecular mechanics (QM/MM) simulations (*2*, *5*): The system is divided into a small QM region modeled with ab initio methods (e.g., substrate and active site of an enzyme) and an MM region (e.g., the remaining protein and solvent molecules) described with an FF. However, the high computational cost associated with an accurate treatment of the QM region and the fact that it is often unclear which atoms need to be included for an adequate description of the process of interest (*6*) may limit the applicability of QM/MM methods.

In recent years, machine-learned force fields (MLFFs) have emerged as an alternative means to execute MD simulations, combining the computational efficiency of traditional FFs with the high accuracy of quantum-chemistry methods (*7*). To construct an MLFF, a machine learning (ML) model is trained on ab initio reference data to predict energies and forces from atomic positions—without the need to explicitly solve the Schrödinger equation outside of the reference data. MLFFs have led to numerous insights, e.g., regarding reaction mechanisms (*8*), or the importance of quantum-mechanical effects for the dynamics of molecules (*9*) and have been successfully applied to MD simulations of small- to medium-sized systems (tens to hundreds of atoms) in gas phase (*10*) and periodic materials (e.g., metallic copper) with millions of atoms (*11*). Despite these successes, applications to large heterogeneous systems, like proteins or other biologically relevant systems, have largely remained elusive, due to the increased complexity of constructing physically informed ML architectures and obtaining reliable reference data for long-range interactions, which are known to play a key role in biomolecular dynamics (*12*, *13*). While the construction of MLFFs for oligopeptides (*14*) and proteins (*15*) has been attempted previously, so far, they have not been demonstrated to yield stable and accurate dynamics over extended timescales (several nanoseconds). A more detailed overview over conventional and MLFFs can be found in section S1.

**Fig. 1.**
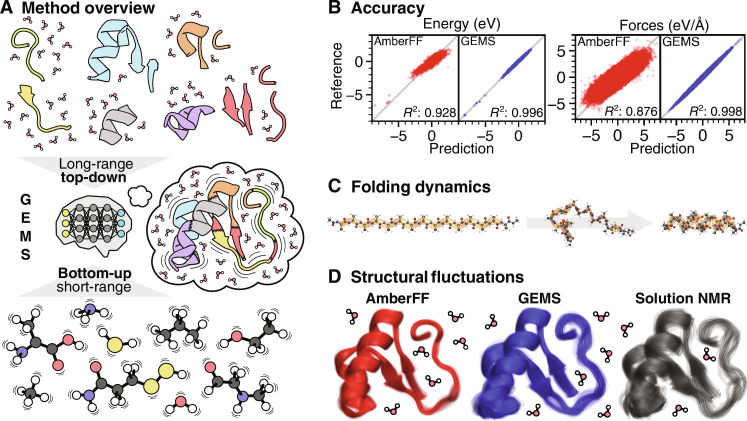
Insights from GEMS simulations. (**A**) Overview of the GEMS method. Different interaction scales on the PES of a large system are learned from a combination of ab initio reference data for top-down and bottom-up fragments. The resulting model is able to accurately reconstruct the PES of the molecular system and then used to study its dynamics. (**B**) Prediction accuracy for energies and forces of AceAla_15_Nme conformations of GEMS compared to AmberFF (*24*) with respect to the PBE0/def2-TZVPP+MBD (*28*, *29*, *44*) reference. Note that AmberFF was not fitted to PBE0/def2-TZVPP+MBD reference data, so a direct comparison can only show qualitative trends. (**C**) GEMS simulations show that the folding of AceAla_15_Nme from a fully extended structure (FES) (left) to a helical conformation (right) at 300 K in gas phase occurs via intermediate conformations characterized by hydrogen bonding between backbone atoms of adjacent residues (middle). (**D**) Overlay of representative conformations (obtained from cluster analysis) sampled during an aggregated 10 ns of NPT dynamics of crambin in aqueous solution at 300 K and ambient pressure (see also fig. S13 for a 360° view of crambin highlighting the interactions relevant for its three-dimensional structure). Simulations with GEMS (blue) lead to greater structural fluctuations compared to AmberFF (red), indicating that the protein is more flexible. For comparison, 20 low energy water refined structures of crambin in dodecylphosphocholine micelles based on NMR measurements (gray) are shown as well (*47*). To allow a quantitative comparison, structures should be modeled with GEMS instead of a conventional FF when interpreting the NMR results.

This work proposes a general approach to constructing accurate MLFFs for large-scale molecular simulations (GEMS). On the basis of the divide-and-conquer principle, MLFFs for large heterogeneous systems are trained on molecular fragments of varying size, which are still amenable to electronic-structure calculations. These fragments do not form a partition of the larger system; rather, they can be overlapping pieces, or even just be structurally related to the original system. The fragments are not used directly when evaluating the MLFF, but only during the training process to learn the relevant physicochemical interactions present in the larger system. From these fragment data (which include water or solvent molecules), the ML model infers to recompose the original system and is able to predict the full potential energy surface (PES) including interactions with solvent, which allows GEMS to successfully address the long-standing challenge of biomolecular simulations at ab initio quality (Fig. 1A). As such, GEMS refers to the general principle of running molecular simulations with MLFFs constructed in this fashion (see also fig. S21 for a schematic depiction).

While MLFFs can successfully learn local chemical interactions from small molecules (*16*), a sufficient number of larger fragments are needed to learn long-range effects necessary to generalize to larger systems and achieve high prediction accuracy (0.450 meV/atom for energies and 36.704 meV/Å for forces) with respect to the ab initio ground truth. Here, we rely on the recently proposed SpookyNet architecture (*17*), which models dispersion and electrostatics explicitly by embedding physically motivated interaction terms into the ML architecture and learning their parameters from reference data. We note that the SpookyNet model is not the first to explicitly model long-range electrostatics, and other models follow similar approaches (*18*–*21*). In addition, an empirical term for short-ranged repulsion between atomic nuclei increases the robustness of the model for strong bond distortions. SpookyNet also includes a mechanism to describe effects like nonlocal charge transfer, which other MLFFs [with some exceptions (*22*)] are typically unable to. Together, these components enable the model to generalize to larger molecules when trained on appropriate reference data. Crucially, this allows GEMS to account for cooperative, long-range effects, which is difficult or impossible for conventional FFs. While extensive reference data for small fragments are mainly used to learn a robust “baseline” representation of short-ranged interactions, additional larger fragments allow GEMS to also capture long-range interactions and the interplay between different interaction scales. In the same manner, solvent effects can also be included (by explicitly describing the interaction with solvent molecules). We demonstrate that GEMS can learn to accurately model large-scale phenomena, such as cooperative polarization effects, from such fragment data, achieving close agreement to the ab initio ground truth.

However, ultimately, the quality and reliability of an MLFF should be judged by its predictions of experimental measurements—for example, we show that GEMS is able to quantitatively reproduce experimental results regarding the helix stability of polyalanine systems at different temperatures and correctly describe the terahertz infrared (IR) vibrational spectrum of a solvated 46-residue protein (crambin), which is extremely difficult to achieve using traditional empirical FFs that do not account for collective many-body interactions and therefore yield large-scale vibrational modes that are qualitative at best, typically giving a smear-out of peak structure and an exaggeration of amplitude over the 25 to 150 cm^−1^ spectral region (*23*).

GEMS is applied to MD simulations of model peptides and the 46-residue protein crambin in aqueous solution with 8205 explicit water molecules (>25,000 atoms). When comparing to conventional FFs, such as AMBER99SB-ILDN (*24*) (AmberFF), GEMS approximates energies and forces computed from density functional theory much more closely (Fig. 1B). Our findings reveal previously unknown intermediates in the folding pathway of polyalanine peptides (Fig. 1C) and a dynamical equilibrium between α- and 3_10_-helices. In the simulations of solvated crambin, GEMS indicates that protein motions are qualitatively different, with much smoother PESs and softened vibrations when compared to computations with a conventional FF (Fig. 1D), showing contrasting short and long timescale dynamics. Low-frequency vibrational modes largely determine the free energy of proteins (*25*); hence, our results suggest that simulations at ab initio accuracy may be necessary to fully understand dynamic processes in biomolecules.

## RESULTS

### MLFFs for large systems trained on diverse chemical fragments

**Fig. 2.**
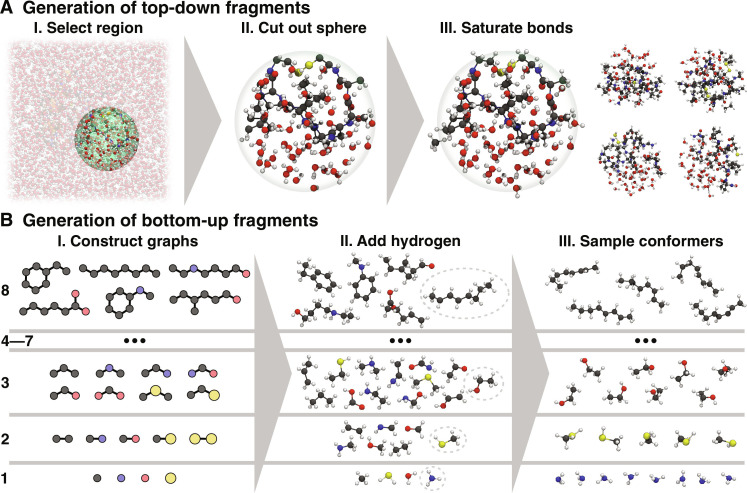
Generation of top-down and bottom-up fragments. (**A**) Top-down fragments are generated by cutting out a spherical region around an atom (including solvent molecules) and saturating all dangling bonds (the right side shows four top-down fragments generated from different regions). They are crucial for learning weak but long-range interactions, which are important for the dynamics of large systems. (**B**) Bottom-up fragments are generated by constructing chemical graphs consisting of one to eight nonhydrogen atoms (not all possible graphs are shown). The graphs are then converted to three-dimensional structures by adding hydrogen atoms. Because of their small size, multiple ab initio calculations for many different conformers of each generated structure can be performed, allowing extensive sampling of the PES, which is necessary for training robust models.

We start by generating reference data for smaller molecular fragments to train an MLFF, where the learned model accurately reflects the full large system. There are several strategies to achieve this goal. On the one hand, the model needs to be able to learn all relevant interactions that are necessary to reconstruct a complete and accurate picture of the system of interest from the fragment data. This is important to capture weak, but long-range interactions, which collectively dominate, e.g., relative energy differences of different conformations of large molecules. On the other hand, it is necessary to prevent “holes” in the PES (*18*)—regions with low potential energy corresponding to unphysical structures, e.g., featuring unnaturally large or short bond lengths. The existence of holes in the PES prevents stable MD simulations, because long trajectories eventually may become trapped by such artefacts and behave unphysically (*26*). To achieve both requirements, we propose the use of two complementary methods to construct fragments, which allow models to learn different aspects of the PES of large systems. The first method follows a top-down approach, where fragments are constructed by “cutting out” spherical regions of the system of interest, which also includes solvent molecules in the condensed phase (Fig. 2A) (*27*). By including solvent molecules in the generated fragment data, the MLFF can learn to treat solvent effects explicitly from reference data. Any dangling bonds resulting from cutting through covalent bonds are saturated with hydrogen atoms or by including a limited number of atoms beyond the cutoff radius (see Materials and Methods for details). The fragments are chosen as large as possible to sample important long-range effects, but still small enough such that reference energies and forces computed with quantum chemistry methods are accessible in a reasonable time. As our tests on polyalanine systems demonstrate (see below), the top-down fragments we choose are sufficiently large for the systems studied in this work. Although any generated top-down fragments are system-specific (except for possible structural similarities to other systems), the method to obtain them is general and can be applied to any large condensed phase system. Further, generated top-down fragments may still be used as training data for learning in new systems.

To train robust models, the top-down fragments are enriched by smaller bottom-up fragments, for which atomic forces for many different conformations can be calculated. Starting from single atoms, molecules similar to local bonding patterns of the system of interest (*16*) are systematically constructed by growing chemical graphs in a bottom-up fashion (Fig. 2B) (missing valencies are filled with hydrogen atoms, see Materials and Methods for details). By limiting the size of these fragments, it is possible to sample many different conformations, allowing models to learn the effects of strong distortions in local structural patterns, which is key to preventing holes in the PES. As a result, the combination of bottom-up and top-down fragments enables learning accurate and robust MLFFs for large systems.

“Accurate” in this context refers to the ability of the MLFF to reproduce the chosen reference method. The “true accuracy” of the MLFF (and thus also the GEMS method itself), i.e., its ability to capture the physics of a particular system of interest, is tied to the accuracy of the underlying reference method chosen to compute the training data. Here, we choose PBE0+MBD (*28*, *29*) as reference method. It explicitly includes long-range dispersion interactions, yet is sufficiently efficient to perform reference calculations for the larger top-down fragments. This level of theory has been shown to provide an accurate and reliable description in excellent agreement with high-level quantum chemistry methods and experiment for, e.g., polypeptides (*30*, *31*), supramolecular complexes (*32*), and molecular crystals with and without water (*33*, *34*), which show very similar bonding patterns as the biomolecular systems studied here. Additionally, PBE0+MBD has been found to be well suited for modeling interactions of proteins in water (*13*, *35*).

To summarize, the training data for GEMS consist of a large number of small “general” fragments (2,713,986 structures), which can be shared for a wide class of chemical systems (in this work: peptides/proteins in gas phase and aqueous solution covering interatomic distances from below 1 Å to about 12 Å), and a small number of large system-specific fragments (covering interatomic distances up to 18 Å). For example, for training GEMS for crambin, 5624 additional top-down fragments are used (see also fig. S25 for a histogram showing the size distribution of fragments for the crambin training data and fig. S26 for an overview over the distribution of pairwise distances). In total, the dataset used in this work to build MLFF models amounts to about 60 million atomic forces, ranging from 650,000 forces for sulfur to 37.6 million forces for hydrogen. The constructed fragments also contain substantial information about water interacting with protein fragments, with about 5 million water molecules in total.

### Polyalanine systems

We apply GEMS to predict the properties and dynamics of several peptides consisting primarily of alanine. These are popular model systems for proteins and well studied both theoretically and experimentally. Further, by limiting the number of residues, it is still possible to perform electronic-structure calculations for the full system. Thus, the predictions of an ML model trained only on fragment data can be directly compared to reference calculations, which allows to verify the ability of GEMS to reconstruct the properties of larger systems from the chemical knowledge extracted from smaller molecules.

**Fig. 3.**
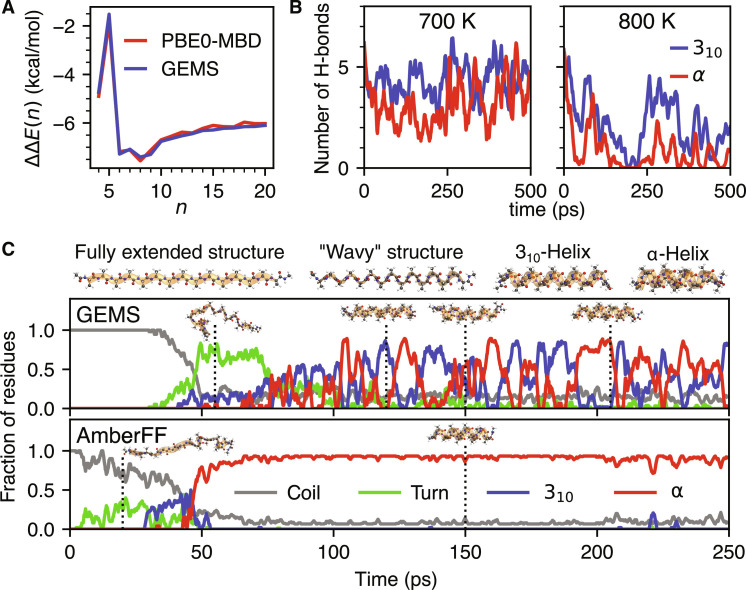
Accurate simulations of polyalanine systems with GEMS. (**A**) Relative stabilization of the α-helical conformation of AceAla*_n_*Lys + H^+^ per added alanine residue. Shown here is the double difference ΔΔ*E*(*n*) = Δ*E*(*n*) − Δ*E*(*n* − 1), where Δ*E*(*n*) = *E*_α_(*n*) − *E*_FES_(*n*) is the relative energy of the α-helical conformation and the FES of AceAla*_n_*Lys + H^+^ in gas phase. The prediction of GEMS (blue) is compared to ab initio reference data computed at the PBE0+MBD (*28*, *29*, *44*) level of theory. (**B**) Number of α- and 3_10_-helical H-bonds during MD simulations of helical AceAla_15_Lys + H ^+^ in gas phase at 700 K and 800 K with GEMS. The sharp drop in the number of H-bonds in the dynamics at 800 K indicates the formation of a random coil (see fig. S12A for an extended version of this figure with a greater range of temperatures). (**C**) Secondary structural motifs determined by STRIDE (*79*) along typical folding trajectories of AceAla_15_Nme at 300 K in gas phase. Dotted vertical lines indicate the temporal position of the shown snapshots. The trajectory computed with GEMS (top) folds via a distinct “wavy” intermediate (classified primarily as “turn”) and settles into a dynamic equilibrium between 3_10_- and α-helices. In contrast, the trajectory computed with the AmberFF (bottom) folds more directly and then stays primarily α-helical (see fig. S9 for an analysis of additional trajectories).

As a first test case, we consider the cooperativity between hydrogen bonds in polyalanine peptides capped with an N-terminal acetyl group and a protonated lysine residue at the C-terminus (AceAla*_n_*Lys + H ^+^). In α-helices, the local dipole moments of hydrogen bonds formed between backbone peptide groups are aligned, leading to a cooperative polarization effect (*36*). Thus, the relative stabilization energy of an α-helix compared to a fully extended structure (FES) fluctuates nontrivially with helix length and is a challenging prediction task. We find that GEMS closely agrees with the reference ab initio method, demonstrating that large-scale effects can be learned effectively from fragment data (Fig. 3A).

Alanine-based peptides have a strong tendency to form helical structures. While short isolated helices are only marginally stable in solution, AceAla_15_Lys + H^+^ is known to form stable helices in gas phase. Experimental results suggest that AceAla_15_Lys + H^+^ remains helical up to temperatures of ∼725 K (*37*), allowing a direct comparison with theoretical predictions. By running GEMS simulations at different temperatures, we confirm that the peptide remains primarily helical up to 700 K, but forms a random coil at 800 K (see movie S1). An analysis of the formed hydrogen bonds reveals that the average number of α-helical hydrogen bonds decreases with increasing temperature (see fig. S12A), while the number of 3_10_-helical hydrogen bonds remains almost constant until a sudden drop at 800 K (see Fig. 3B). This agrees with results from ab initio MD simulations at the PBE+vdW level (*38*), where a similar relationship between temperature and the stability of different kinds of hydrogen bonds was found. The long-range interactions learned from top-down fragments seem to be crucial to reproduce the experimental results, as a model that was only trained on bottom-up fragments predicts reduced thermal stability (see fig. S12B).

To investigate whether there are fundamental differences between GEMS and dynamics simulations performed with conventional FFs, we study the room temperature (300 K) folding process of a pure polyalanine peptide capped with an N-terminal acetyl group and a C-terminal N-methyl amide group (AceAla_15_Nme) in gas phase. Starting from the FES, MD simulations with GEMS suggest that AceAla_15_Nme has a strong tendency to form H-bonds between peptide groups of directly adjacent residues within the first ∼100 ps of dynamics. The formed arrangements exhibit a “wavy” structure and ϕ and ψ backbone dihedral angles of ∼0° and ∼0°, which lie in a sparsely populated region of the Ramachandran plot. These intermediates are typically short-lived with lifetimes of ∼25 to 50 ps and fold readily into helical configurations via a characteristic twisting motion. There is still some controversy between theoretical and experimental results regarding the predominance of different helical conformations (*39*). We find that there may be cases where no single motif is preferred: Once a helix is formed, its structure fluctuates between pure α- and 3_10_-helices, as well as hybrids of both helix types (see movie S2 for a complete folding trajectory). A 10-ns trajectory of the helical state suggests a dynamical equilibrium with a ∼38/62% mixture of α- and 3_10_-helices. Such a dynamical coexistence of α- and 3_10_-helices has already been observed experimentally in alanine-rich peptides (*40*, *41*) and can be assessed for the specific example of AceAla_15_Nme in future experiments using nuclear magnetic resonance (NMR) or ultraviolet (UV)/IR spectroscopies on polyalanine folding under “clean room” gas-phase conditions. In contrast, MD simulations with the AmberFF yield qualitatively different results, suggesting that a more rigid and primarily α-helical configuration is formed from the FES without distinct structural intermediates (see Fig. 3C). Additionally, we also investigated the dynamics with the CHARMM27 (*42*) and GROMOS96 53A5 (*43*) FFs (see fig. S11 for representative trajectories). While the dynamics with CHARMM27 are comparable to those of AmberFF (apart from typically folding slightly later during the dynamics), we could not observe helix formation when using GROMOS96 53A5 at all. However, the “wavy intermediate” observed in the GEMS simulations (where this structure seems to be metastable) is readily formed almost instantly in many GROMOS trajectories, but does not fold to a helix subsequently and instead is stable over hundreds of picoseconds. The partial agreement of different classical FFs with the GEMS trajectory can thereby be understood as the parametrization process of the individual FFs imposing a select, limited set of correct constraints. As a result, each FF correctly captures certain aspects (such as formation of the wavy intermediate or folding into an α-helix) but fails to correctly reproduce all features due to the limited flexibility of the fixed FF energy functional. Altogether, the ab initio accurate GEMS simulations provide a concrete prediction of a dynamical coexistence of α- and 3_10_-helices in AceAla_15_Nme, which is in line with experimental observations for alanine-rich polypeptides, but is not predicted by conventional FFs. It is important to point out that conventional FFs are usually parametrized for simulations in solvent, not in the gas phase. As such, their performance in gas phase is not necessarily an indicator for their performance in solution. The results shown here for different conventional FFs are meant to emphasize that differences in parametrization can lead to substantially different dynamics, which all differ qualitatively from the dynamics predicted by GEMS. We note in passing that many other FFs are available in the literature. However, most of them are based on the same restricted functional form for the bonded interactions. A comprehensive assessment of different generations of empirical FFs goes beyond the scope of our work.

For completeness, we also investigate trajectories of AceAla_15_Nme simulated with a GEMS model that was only trained on bottom-up, but not top-down, fragments. In this setting, we observe no helix formation on the investigated timescale; instead, AceAla_15_Nme typically stays structurally close to the FES during the dynamics, only rarely forming partial loop motifs (see fig. S10). This suggests that the inclusion of top-down fragments is crucial to correctly describe the folding process and is consistent with the results obtained for the thermal stability of AceAla_15_Lys + H^+^, where a model trained without top-down fragments predicts diminished stability of the folded state (see also fig. S12B).

As a final test for the accuracy of GEMS, we compare the predictions of the ML model to ab initio data computed at the PBE0/def2-TZVPP+MBD (*28*, *29*, *44*) level of theory. To this end, we use 1554 and 1000 AceAla_15_Nme structures sampled from densely and sparsely populated regions (rare events) of the configurational space visited in 100 aggregated 250-ps MD trajectories (25 ns total) in the NVT ensemble at 300 K simulated with GEMS (see section S4 for details). We find that predicted energies and forces are in good agreement with the reference values in both cases. For energies and forces, correlation coefficients are *R*^2^ = 0.996 and *R*^2^ = 0.998, respectively, and mean absolute errors (MAEs) are 0.450 meV/atom and 36.704 meV/Å. Again, we find that the inclusion of top-down fragments during training is crucial for high accuracy, as prediction errors for a model trained only on bottom-up fragments are much larger (see fig. S8). For completeness, we also compare predictions with the conventional AmberFF (*24*) to the ab initio reference. Although AmberFF is not fitted to reproduce energies and forces from density functional theory (DFT) calculations, its predictions display correlation coefficients of *R*^2^ = 0.928 (for energy) and *R*^2^ = 0.876 (for forces). Nonetheless, the MAEs are much larger at 2.274 meV/atom and 329.328 meV/Å (distributions of predicted and reference energy values were shifted to have a mean of zero before computing MAEs in both cases such that constant energy offsets between different methods do not influence the results). Although a quantitative comparison between GEMS and AmberFF in this context is not meaningful, as a qualitative trend, we observe that predictions with GEMS reproduce the reference across the whole range of values without the presence of a single outlier, whereas the AmberFF systematically under- and overpredicts small and large energy values, respectively (see Fig. 1B). These findings show that GEMS gives accurate predictions even for rare configurations and the simulated MD trajectories are essentially ab initio quality (see figs. S6 and S7 for a more detailed analysis of correlations within the different subsets of configurations). This comparison between GEMS and AmberFF also suggests that reproducing relative energies of different protein conformations is an easier task than accurately capturing atomic forces that drive the biomolecular dynamics.

### Crambin

**Fig. 4.**
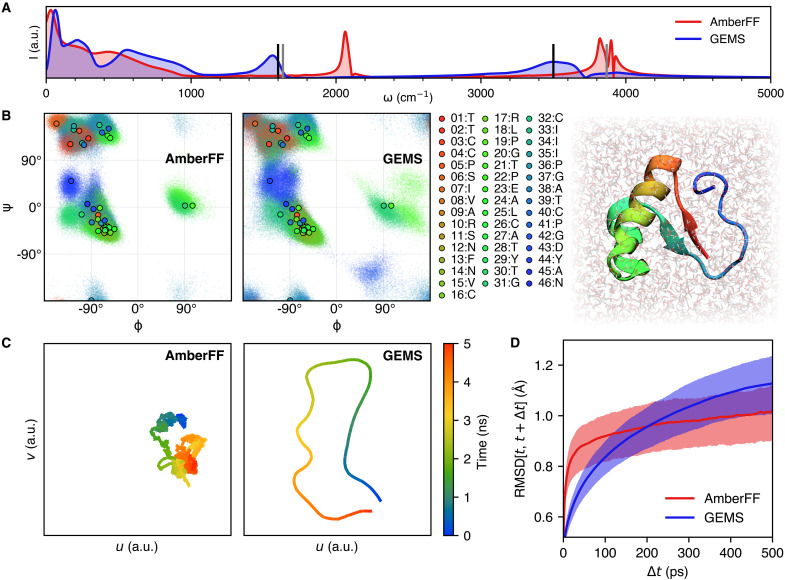
Analysis of dynamics simulations of crambin in aqueous solution. (**A**) Power spectrum of crambin in water obtained from 125 ps of dynamics computed with the AmberFF (without any constraints on bonds to hydrogen) and GEMS. In the GEMS spectrum, peaks associated with bending and stretching vibrations of water molecules are closer to experimentally expected values at around ∼1600 cm^–1^ and ∼3500 cm^–1^ (*46*) (vertical black lines). For reference, we also show the gas-phase harmonic peaks calculated at the PBE0+MBD level of theory (vertical gray lines). (**B**) Ramachandran map for crambin (color-coded by residue number). The scatter shows the (ϕ, ψ)-dihedral angles sampled during an aggregated 10 ns of dynamics; points with black outline show values of the crystal structure (*70*) for reference. Dynamics with GEMS (right) generally sample a broader distribution compared to AmberFF (left), indicating that the protein is more flexible. (**C**) Two-dimensional Uniform Manifold Approximation and Projection (UMAP) (*80*) projection of the path through conformational space sampled during a 5-ns trajectory of crambin in aqueous solution. Compared to the trajectory computed with the AmberFF, dynamics with GEMS sample a wider distribution and are less likely to revisit previously visited regions of conformational space. (**D**) Distribution of RMSDs (excluding hydrogen atoms) between conformations sampled at times *t* and *t* + Δ*t*. Solid lines depict the mean, whereas the shaded region indicates the area between the 25th and 75th percentiles. Dynamics with the AmberFF (red) show larger structural fluctuations on short timescales, whereas fluctuations on longer timescales are larger for dynamics computed with GEMS (blue).

GEMS enables accurate molecular simulations in the condensed phase. The 46-residue protein crambin in aqueous solution (25,257 atoms) is chosen as a model system. Crambin contains 15 of the 20 natural amino acids and forms common structural motifs such as β-sheets, α-helices, turns/loops, and disulfide bridges. To assess qualitative differences between simulations with a conventional FF (here, the AmberFF is chosen) and GEMS, we consider the power spectrum (*45*) computed from 125 ps of dynamics at a temporal resolution of 2.5 fs (Fig. 4A) (no constraints were used for bonds to hydrogen atoms in these simulations). The power spectrum is related to the internal motions of the system and reveals the dominant frequencies of molecular vibrations, which are influenced by the atomic structure and characteristic for the presence of certain functional groups. In comparison to the results obtained from the dynamics with a conventional FF, peaks in the power spectrum computed with GEMS are shifted toward lower wave numbers and lie close to the frequency ranges expected from measured IR spectra. For example, the dominant peaks above 1000 cm^–1^ correspond to bending and stretching vibrations of water molecules, which are experimentally expected at around ∼1600 cm^–1^ and ∼3500 cm^–1^, respectively (*46*), which is consistent with the GEMS spectrum. In contrast, the corresponding peaks for the conventional FF are blue-shifted several hundreds of wave numbers. Additionally, peaks in the GEMS spectrum are broader, indicating that the frequencies of characteristic vibrations are influenced stronger by intermolecular interactions, hence broadening their frequency range. Long-range interactions may particularly influence slow protein motions, i.e., the low-frequency parts of the power spectrum, where notable differences between GEMS and AmberFF can be observed.

**Fig. 5.**
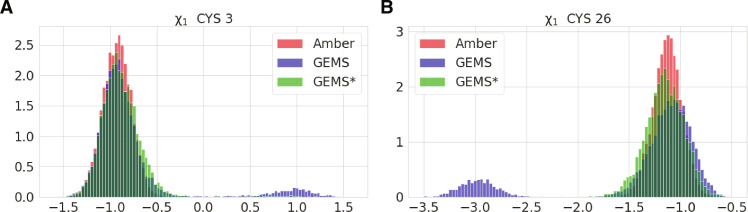
Comparison of dihedral angles of disulfide bridges under different FFs. (**A**) Residue 3. (**B**) Residue 26.

**Fig. 6.**
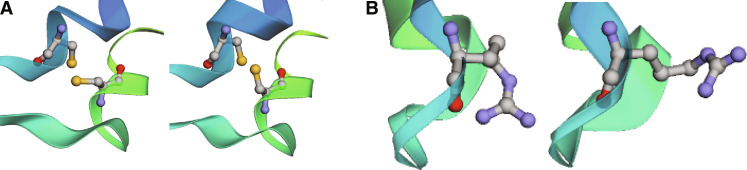
Cysteine/arginine residues in crambin. (**A**) Different torsion angles χ_1_ in CYS3, as observed in the GEMS trajectory. (**B**) Different ARG17 configurations, as observed in the GEMS trajectory.

Similar to the results for AceAla_15_Nme, we find that in comparison to simulations with the AmberFF, crambin is more flexible in GEMS simulations (Fig. 1D). Qualitatively, the increased flexibility seems to agree more closely to structures modeled from NMR spectroscopy measurements (see Fig. 1D), but a direct comparison of root mean square deviation (RMSD) values along GEMS and AmberFF trajectories reveals that both simulation types agree similarly well with the structural ensemble (see figs. S22 and S23). The direct comparison to the structures published in (*47*) should be regarded with caution, as the structural ensemble is not obtained by a direct experimental measurement, but rather fitted to experimentally obtained distance constraints with a conventional FF, which may introduce notable bias. The increased flexibility is also indicated by a Ramachandran map of the backbone dihedral angles of crambin (Fig. 4B), which shows that a wider range of values is sampled in simulations with GEMS (backbone bond length distributions are, however, comparable between both simulations; see fig. S15). Similar results can be observed in Fig. 5, where the GEMS simulation shows an additional mode for the torsion angle in two of the six cysteine residues that form disulfide bridges, and in fig. S18, which shows that the GEMS trajectories contain more modes of the distributions for the four torsion angles in ARG17. An illustration of different configurations is shown in Fig. 6.

This becomes even more apparent by projecting the trajectories into a low-dimensional space that allows a direct visualization of the path taken through conformational space (Fig. 4C). However, a time-resolved analysis of the trajectories reveals that structural fluctuations with GEMS are only larger on timescales in excess of ∼200 ps (Fig. 4D). On shorter timescales on the other hand, the trend is reversed. Despite the fact that fluctuations in GEMS trajectories seem to be growing on larger timescales, we find that the overall structure stays close to the folded state at all times (see fig. S24), i.e., we do not observe any signs of early unfolding. We can also see the timescale dependence directly on the torsion angles: When forming a moving average over 100 time steps, short time fluctuations cancel out more for the AmberFF trajectories, which leads to sharper peaks in the distributions. As an example, fig. S20 shows the distribution of the torsion angles for the other four cysteine residues. While the original distributions (on the left) do not differ much between GEMS and AmberFF, the distributions of the averaged angles (on the right) show a much sharper peak for AmberFF. The same can be observed in fig. S19 for ARG10. This suggests that there are qualitative differences between simulations with conventional FFs and GEMS on all timescales.

**Fig. 7.**
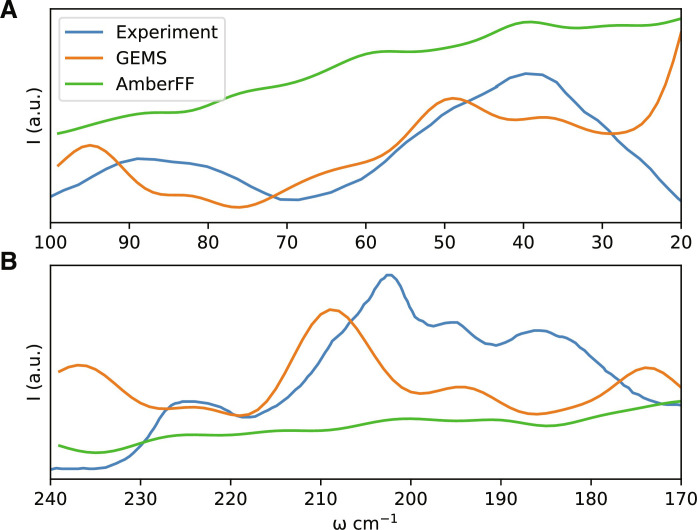
IR spectrum of crambin on the terahertz timescale. (**A**) Frequency range from 20 to 100 cm^−1^. (**B**) Frequency range from 170 to 240 cm^−1^. Good agreement between GEMS and the experimental spectrum (*48*) is found, whereas the spectrum computed from simulations with AmberFF is smooth and largely featureless.

To investigate whether the difference in structural fluctuations has a direct effect on experimental observables, we compute terahertz timescale IR spectra of solvated crambin simulated with GEMS and AmberFF to experimental measurements for a partially solvated crambin sample (*48*). The terahertz spectrum corresponds to slow dynamics of the folded protein in solvent, and hence, it is sensitive to the correct description of long-range interactions. We find that GEMS is able to reproduce most experimentally observed features rather well (see Fig. 7), whereas the spectrum computed from AmberFF simulations lacks the distinctive features of the experimental spectrum and is largely featureless over the range for which experimental data are available, consistent with the vibrational power spectrum shown in Fig. 4A. We remark that one cannot expect quantitative agreement with experiment, given that the solvent in the experimental system was a mixture of water and organic salts, while the simulations were run in pure water, with dielectric screening from partial solvent then recalculated post hoc (see Materials and Methods for details).

In addition, we also compare the crambin dynamics observed with GEMS to those of a model that was only trained on (general) bottom-up fragments, but not on (system-specific) top-down fragments (referred to as GEMS* in the following). While a visual inspection of the GEMS* trajectories suggests no marked differences to the regular GEMS model, a detailed analysis reveals that, while being qualitatively similar overall, some regions of the Ramachandran map are less frequently visited and appear closer to the observations for AmberFF (see fig. S17A). Further, the additional modes (compared to the AmberFF trajectory) in the distribution of the cysteine torsion angles (that the GEMS trajectory showed) vanish for GEMS* (see Fig. 5). Also, the distribution of torsion angles for ARG17 in GEMS* lies somewhere in between the observations for GEMS and AmberFF (see fig. S18). Similarly, while structural fluctuations on short timescales agree between GEMS and GEMS*, the long timescale fluctuations of GEMS* are smaller than those for GEMS and instead closer to those observed for AmberFF (see fig. S17B). As such, it appears that long-range effects learned from top-down fragments are crucial for describing the crambin dynamics on long timescales.

### A model for understanding the different dynamics of crambin

Reasons for the observed qualitative differences between AmberFF and GEMS trajectories must be related to differences in the PESs. AmberFF is a conventional FF, and as such, models bonded interactions with harmonic terms. Consequently, structural fluctuations on small timescales are mostly related to these terms. Intermediate-scale conformational changes as involved in, for example, the “flipping” of the dihedral angle in the disulfide bridges of crambin, on the other hand, can only be mediated by (nonbonded) electrostatic and dispersion terms, because the vast majority of (local) bonded terms stay unchanged for all conformations. On the other hand, GEMS makes no distinction between bonded and nonbonded terms, and individual contributions are not restricted to harmonic potentials or any other fixed functional form. Consequently, it can be expected that large structural fluctuations for AmberFF always correspond to “rare events” associated with large energy barriers, whereas GEMS dynamics arise from a richer interplay between chemical bonds and nonlocal interactions.

**Fig. 8.**
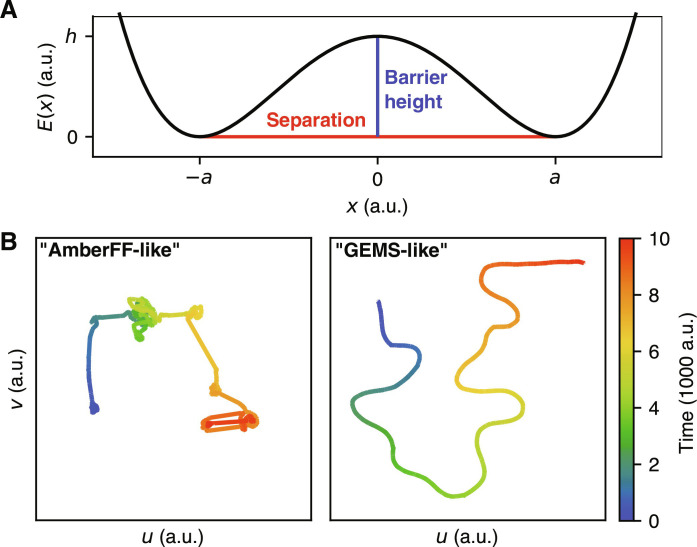
Simplified model for PESs with different dynamical properties. (**A**) One-dimensional double-well potential showing the effects of the separation parameter *a* (large values of *a* correspond to large possible conformational changes) and the barrier height *h*. (**B**) Low-dimensional projections (similar to Fig. 4C) of trajectories on a PES where large conformational changes are always associated with high energy barriers (“AmberFF-like”) and a PES where large conformational changes may be associated with small energy barriers (GEMS-like).

To test this hypothesis, we introduce a simplified model of a high-dimensional PES based on superposed one-dimensional oscillators confined to a double-well potential. The potential energy is given by

$$E\left(x\right)=\sum_{i=1}^{N}‍{\left(\frac{h_{i}}{a_{i}}\right)}^{4}{\left(x_{i}^{2}-a_{i}^{2}\right)}^{2}$$ (1)

where *h_i_* is the barrier height between two minima, 2*a_i_* is their separation, and *x_i_* is the coordinate of oscillator *i* (see Fig. 8A). The vector **x** = [*x*_1_…*x_N_*]^⊺^ is the “configuration” of the *N*-dimensional model system. We then simulate the trajectory **x**(*t*) with Langevin dynamics, which couples the oscillator modes to a shared heat bath that allows energy transfer between them. This setup constitutes a simplified model of PESs with different qualitative properties. We find that for a system where large conformational changes (*a* is large) are always associated with high energy barriers (*h* is large, roughly ∼4 times larger than for small conformational changes), low-dimensional projections of **x**(*t*) resemble those of crambin simulated with the AmberFF. On the other hand, if large conformational changes are also possible with low energy barriers, the projections resemble those of the GEMS trajectory (see Figs. 4C and 8B). All computational details for these experiments are given in Materials and Methods. These results suggest that GEMS simulations substantially enhance large-scale structural transitions between distant conformations, which is in agreement with the flexibility of crambin observed in NMR experiments (see Fig. 1D) (*47*). These results are also consistent with (i) the much increased flexibility of polyalanine helices observed with GEMS in comparison to the essentially rigid dynamics obtained in AmberFF simulations (see Fig. 4C) and (ii) the inability of traditional FFs to reproduce enhanced structural fluctuations of mobile protein regions observed in NMR experiments (*49*).

We would like to reiterate that this analysis addresses the PES topology of crambin in the folded state. As such, the results shown in Fig. 8 and discussed above concern larger-scale corrugation rather than global PES barriers (i.e., flexibility within structural basins rather than folding events). On the basis of the increased smoothness and flexibility found in GEMS, we expect that the correct, ab initio accurate treatment would predict a broader ensemble of folding pathways characterized by more collective, low-frequency rearrangements. This, however, does not necessarily affect the overall timescales of folding. Increased flexibility and a wider transition path ensemble accompanied by lower-frequency (slower) dynamics can preserve the (relative) phase space volumes of PES minima and the transition state (ensemble). With relative phase space volumes being the key quantity in standard reaction rate theory, the topological changes may thus not affect the overall folding rate. On the other side, conventional FFs are typically designed to reproduce properties such as folding rates to the largest extent possible within the fixed form of the classical energy functional. Simply reproducing the correct timescales, however, does not necessarily entail the correct dynamics or folding pathway, where it is known that different choices of conventional FFs produce substantially different results (*50*).

## DISCUSSION

Modeling quantum-mechanical properties of large molecules is an outstanding challenge, and it holds promise for broad application in chemistry, biology, pharmacology, and medicine. We have developed a general framework for constructing MLFFs—GEMS—for large molecular systems such as proteins by learning from ab initio reference data of small(er) fragments without the need to perform electronic-structure calculations for a whole protein—as the latter would constitute a computationally impractical task. The proposed divide-and-conquer strategy using a library of ∼3 million DFT+MBD computations on fragments and using an ML model that incorporates physical constraints and long-range interactions allows to efficiently construct MLFFs that accurately reflect the quantum-mechanical energies and atomic forces in large molecules. An interesting insight of our ab initio accurate simulations is that proteins seem to be substantially more flexible than previously thought. These molecular fluctuations and associated low-frequency vibrations are expected to strongly contribute to dynamical processes such as in biomolecules (*51*).

While our work focuses exclusively on the study of peptides and proteins, the proposed framework can be applied to any atomic system too large to study with ab initio methods. We find that even small polyalanine peptides display qualitatively different dynamics when simulated with GEMS in comparison to dynamics with conventional FFs. For example, GEMS simulations suggest that the folding of AceAla_15_Nme from the FES to a helical conformation occurs via short-lived intermediates characterized by hydrogen bonding between peptide groups of adjacent residues. Once a helix is formed, its structure fluctuates between 3_10_- and α-helices in a dynamical equilibrium. This is in stark contrast to simulations with a conventional FF, where the peptide forms a rigid α-helix without visiting a common intermediate. Future NMR or UV/IR spectroscopy experiments could confirm or disprove our predictions for polyalanine helices. These results are reminiscent of the first MD study of a protein (*52*), which showed that proteins are less rigid than previously thought (*53*). The current findings, already alluded to above, indicate that proteins might be even more flexible, and our simulations of crambin suggest that the general trend observed for peptides in gas phase also holds for proteins in solution. In particular, crambin samples a larger conformational space in GEMS simulations and its backbone dihedral angles have broader distributions. Experiments with a simplified model for the PES suggest that the increased flexibility observed in GEMS simulations is associated with low energy barriers for large conformational changes, whereas with conventional FFs, large fluctuations are always associated with large barriers. This could explain the long-standing disagreement between classical MD and the structural fluctuations of mobile protein groups observed in NMR experiments (*49*). However, structural fluctuations on short timescales are reduced in comparison to simulations with a conventional FF. These observations show that there are qualitative differences in the dynamics of proteins when they are simulated with ab initio quantum-mechanical accuracy.

A promising avenue for future work is to extend GEMS to larger systems and longer timescales, for example, by distributing GEMS simulations over multiple accelerators, which requires nontrivial modifications to the way the MLFF is evaluated. Other possible extensions to GEMS include incorporating nuclear quantum effects, which were demonstrated to substantially change the dynamics of small molecules (*54*). It is likely that similar effects can be observed for larger systems.

Let us discuss some limits of MLFFs when compared to classical MD simulations. Although MLFFs are orders of magnitude more computationally efficient than ab initio calculations, their computational efficiency is lower than that of conventional FFs (as to be expected). For example, simulating a single timestep of NPT dynamics of crambin in aqueous solution on an NVIDIA A100 GPU with GEMS takes roughly ∼500 ms, whereas GROMACS (*55*) only requires ∼2 ms for a single time step with a conventional FF on similar hardware. Consequently, at this moment, GEMS simulations are limited to shorter timescales. In addition, evaluating MLFFs usually requires increased memory compared to conventional FFs, limiting the maximum system size that can be simulated with GEMS. Nonetheless, GEMS allows to simulate several nanoseconds of dynamics for systems consisting of thousands of atoms with ab initio accuracy. Furthermore, GEMS like every other MLFF may lead to unphysical dynamics, if not properly trained [see, e.g., (*26*) for a discussion of such phenomena]. As a rule, MLFF simulations should therefore always be subjected to more scrutiny than results from mechanistic FFs. In particular, the resulting trajectories need to be carefully checked for unphysical bond breaking or formation, or otherwise unphysically distorted conformations, which are prevented in traditional FFs by construction. Nevertheless it should be emphasized again that compared to simulations with a conventional FF, GEMS offers highly improved accuracy as well as enables to study chemical transformations such as the making and breaking of chemical bonds and proton transfer processes.

Another advantage of using accurate MLFFs is the availability of arbitrary derivatives—including the potential to obtain alchemical derivatives (*56*). This may enable the optimization of accessible observables, such as docking/binding energies, with respect to local (nearly isosteric) mutations. In a more conventional approach, MLFFs can be used to describe the effects of mutations via thermodynamic integration as regularly performed with classical FFs (*57*, *58*). Given the incorporation of nonlocality in the present methodology, such analyses could naturally account for longer-range phenomena like (static) allosteric effects and the inherent nonadditivity of interactions known to be relevant for the free energy of binding or stability (*59*). In a similar vein, this may allow to perform sensitivity analyses to identify allosteric hotspots and networks, which have also been speculated to play an important role for the evolutionary aspects of proteins and the biomolecular machinery (*60*). Again, we would like to stress that such studies and the above approaches are not limited to biomolecular systems. They may equally well be applied in materials design, for example, studying and optimizing point defects in solid-state systems as relevant to the design of quantum materials.

**Fig. 9.**
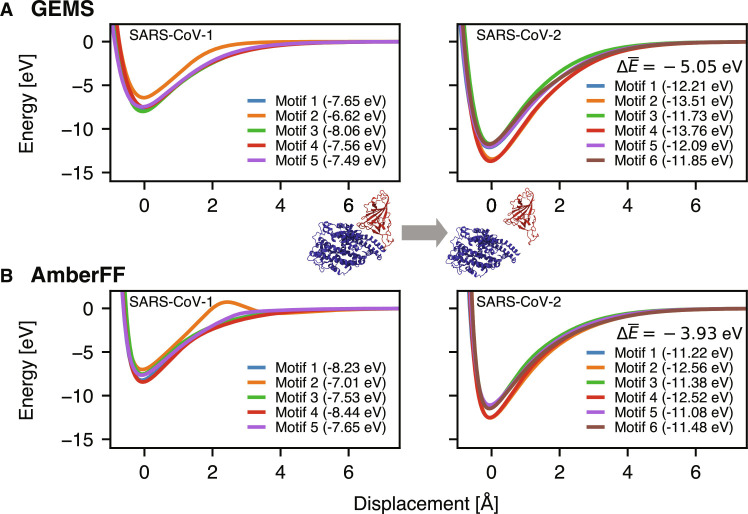
Toward accurate quantum-mechanical protein-protein interactions: Gas-phase binding curves of ACE2 (blue) and RBD of the SARS-CoV spike protein (red). The ACE2 and RBD proteins are displaced along the line connecting their centers of mass relative to their equilibrium position in solution (computed with the AmberFF), keeping their internal structure fixed. Different binding motifs [taken from (*74*)] are distinguished (values in brackets are the maximum well depth for the corresponding motif). All energy values are referenced with respect to infinite separation of ACE2 and RBD. The displayed value $\Delta \bar{E}$ gives the difference in well depth (averaged over all binding motifs) between SARS-CoV-2 and SARS-CoV-1. The $\Delta \Delta \bar{E}$ between AmberFF (**B**) and GEMS (**A**) is −1.11 eV.

Finally, we would like to highlight a promising application of GEMS to modeling protein-protein interactions. Figure 9 shows the binding curves of the angiotensin converting enzyme 2 (ACE2) and the receptor binding domain (RBD) of the spike protein of severe acute respiratory syndrome coronavirus 1 (SARS-CoV-1) and SARS-CoV-2 variants using either AmberFF or GEMS (in gas phase). Here, as expected from experimental evidence (*61*), we observe a stronger binding of SARS-CoV-2 for both the classical FF and GEMS. However, GEMS yields a substantially stronger binding by −1.1 eV. Note that the obtained binding energies were computed for static structures in gas phase and do not account for solvation or entropic effects, nor the presence of dynamic loops at the protein surface, so they cannot be directly compared to experimental binding affinities. However, although these results are preliminary and should not be overinterpreted, they indicate the potential importance of ab initio accuracy when studying interactions between complex biological systems. We therefore would like to stress the high promise of GEMS for enabling quantum-mechanical insight in broad application domains across enzyme and protein chemistry or heterogeneous materials. Although top-down fragments in this work are system-specific, in the future, they may be generated to cover a wider range of systems and enable GEMS simulations with a chemically transferable and size-extensive “universal” MLFF.

## MATERIALS AND METHODS

### Construction of fragment data

#### Bottom-up fragments

The construction of bottom-up fragments follows an approach similar in spirit to the one described by Huang and von Lilienfeld (*16*). Ignoring hydrogen atoms, increasingly large chemical graphs with the same local bonding patterns as the system of interest are constructed until a maximum number of heavy atoms is reached. This is achieved by starting from graphs consisting of a single heavy atom. Larger graphs are constructed by successively adding additional heavy atoms and pruning graphs, which do not appear as substructures in the original system. Once the graphs are constructed, they are converted to bottom-up fragments by saturating all valencies with hydrogen atoms and generating the corresponding three-dimensional molecular structure [e.g., using Open Babel (*62*)]. For all structures, multiple conformers are sampled using either MD simulations at high temperatures or normal mode sampling (*7*). Here, we use the bottom-up fragments for solvated proteins generated in earlier work (*63*). These bottom-up fragments also contain micro-solvated structures with explicit water molecules and structures for bulk water [see (*19*) for details]. Since all similar local structures are covered by the same graphs, the bottom-up fragments optimally exploit any structural redundancies, often resulting in a surprisingly small number of fragments. For example, just 2307 chemical graphs (with a maximum of eight heavy atoms) are sufficient to cover all local bonding patterns appearing in proteins consisting of the 20 natural amino acids, even when considering different protonation states and the possibility of disulfide bridges (*19*).

#### Top-down fragments

Starting from an MD snapshot of the system of interest (sampled from conventional MD simulations, see below and section S3 for more details), all atoms outside a spherical region around a central atom are deleted. The cutoff radius for selecting the spherical region should be chosen as large as possible, but still resulting in fragment sizes for which reference calculations are feasible (here, we choose 8 Å). Then, any resulting dangling single bonds on heavy atoms are saturated with hydrogen atoms. Valencies situated on hydrogen atoms or corresponding to double bonds are eliminated by including the bonded atom in the original system (outside the cutoff). This process is repeated until all valencies are saturated (*27*). By choosing different central atoms, several (partially overlapping) top-down fragments can be constructed from a single configuration of the original system. Since the snapshots from which top-down fragments are constructed are sampled by MD simulations with a conventional FF, structures that are either not well described by the chosen FF or not visited during the dynamics may potentially introduce bias toward certain “interaction motifs” into the training data. Although this is partially alleviated by a thorough sampling of bottom-up fragments, care should be taken that the conventional FF used for sampling is well parametrized. Especially for nonbiological systems, where widely used FF parametrizations are much less common, it might become necessary to sample MD snapshots with, e.g., semi-empirical methods or derive top-down fragments from structures sampled in other ways.

### DFT calculations

DFT reference calculations were performed using the Psi4 software package (*64*) at the PBE0/def2-TZVPP+MBD (*28*, *29*, *44*) level of theory on Google Cloud Platform (GCP). Each fragment was run on an independent Docker container within a cloud compute engine virtual machine. We mostly used n2d-higmem-4 and n2-highmem-4 virtual machine instances with four cores, 32 GB RAM and 768 GB of disk space each, with some larger fragments being manually relaunched on higher-memory machines if they crashed with out-of-memory errors. Execution was parallelized on up to 20,000 CPU cores. Calculations where shut down if they did not complete within 21 days, which was the case for a few outliers, but median execution time per fragment was ~48 hours. For example, of the 2292 crambin fragments, 5% (120) did not finish successfully on an n2-highmem-4 machine, due to machine errors, lack of memory, or because the fragment failed to converge to a meaningful solution. The rest (2172 fragments) all finished within a week, with a median runtime of 47.4 hours (mean: 50.7 hours) (see fig. S16 for the runtime distribution). Only 33 fragments needed more than 4 days of compute to complete. In total, the successful runs required approximately 110,000 compute hours.

### Training the MLF

All MLFFs in this work use the recently proposed SpookyNet architecture [see (*17*) for details]. We use three different trained ML models here: one for the simulations of all polyalanine systems, one for the simulation of crambin in aqueous solution, and one for the gas-phase ACE2/SARS-CoV-1/2 RBD binding curves shown in Fig. 9 (CoV model). The polyalanine and CoV models use the recommended architectural hyperparameters of *T* = 6 interaction modules and *F* = 128 features (*17*). Because of hardware limitations when performing MD simulations for thousands of atoms, the crambin model uses *T* = 3 interaction modules and *F* = 64 features to reduce memory requirements. All models use a short-range cutoff of *r*_srcut_ = 10 *a*_0_ (∼5.29 Å). The crambin and CoV models additionally use a long-range cutoff of *r*_lrcut_ = 20 *a*_0_ (∼10.58 Å) for the computation of the analytical electrostatic and dispersion correction terms included in the SpookyNet energy prediction (to achieve sub-quadratic scaling with respect to the number of atoms). We follow the training protocol described in (*17*) for fitting the parameters to reference energies, forces, and dipole moments; however, the mean squared loss function was replaced by the adaptive robust loss described in (*65*). All models were trained on single NVIDIA V100 GPUs on GCP using the same 2,713,986 bottom-up fragments, and 45,948 (for the polyalanine model), 5624 (for the crambin model), or 129,942 (for the CoV model) top-down fragments. Typical training times are between 1 and 2 weeks, depending on the system. During training, structures were randomly drawn in equal amounts from bottom-up and top-down fragments, i.e., top-down fragments were oversampled to mitigate the imbalance in the numbers of bottom-up/top-down fragments.

### MD simulations

#### Conventional FF

All classical MD simulations have been performed with the GROMACS 2020.3 software package using NVIDIA V100 or A100 GPUs in a Kubernetes system on GCP. Throughout this work, we have used the AMBER99SB-ILDN FF (*24*) for the conventional MD simulations. Standard amino acid definitions have been adapted to accommodate charged Lys + H^+^ termini in accordance with the AMBER99SB-ILDN parametrizations where needed. In the MD simulations of ACE2/SARS-CoV-2 RBD, the binding of the Zn^2+^ cofactor in ACE2 has been described via harmonic restraints to the experimentally determined ligands to avoid potential shortcomings in the description of the metal-ligand interaction. All solvated systems presented in this article or used for sampling representative structures for generating top-down fragments were initially resolvated, optimized to a maximum atomic force of 1000 kJ mol^−1^ nm^−1^, and equilibrated according to the protocol detailed in section S2. Simulations for studying nonequilibrium processes (i.e., the gas-phase folding/unfolding of polyalanine systems) have been started directly from optimized structures with velocities drawn from a Maxwell-Boltzmann distribution at twice the simulation temperature [such that the average kinetic energy during the simulation corresponds to the desired temperature (*66*)]. The gas-phase simulations have thereby been realized in a pseudo–gas-phase setting as proposed and validated in (*66*). All constant temperature MD simulations have been performed using temperature coupling via stochastic velocity rescaling (*67*), and a Parrinello-Rahman barostat (*68*) has been used for NPT simulations. To speed up computations, standard MD simulations involved the commonly used constraint of bonds involving hydrogen with a time step of 2 fs, while the power spectra reported in this work have been obtained from fully unconstrained simulations with a time step of 0.5 fs. The starting structures of polyalanine systems have been generated with the Avogadro software (*69*), and the initial structure of crambin has been taken from Protein Data Bank (PDB) entry 2FD7 (*70*) (resolution, 1.75 Å) of a chemically synthesized mutant of crambin, where we used PyMOL (*71*) to remodel mutated residues to match the wild-type sequence (SER11 and VAL15). This starting structure was chosen because of its favorable validation metrics (e.g., clash score). For completeness, we compared the structure of crambin (after solvation and subsequent minimization with GROMACS) when choosing higher-resolution entries from the PDB [1EJG (*72*) and 3NIR (*73*)] as initial structure instead. The obtained structures are virtually identical with RMSDs below 1 Å (see fig. S14). Our simulations of the ACE2/SARS-CoV-1/2 RBD complex have been initiated from a set of representative conformations as identified in (*74*) or pointwise mutations thereof. Currently available experimental results on the mutations present in the β, γ, δ, and ϵ variants of SARS-CoV-2 do not indicate considerable structural changes to the spike RBD. After partial relaxation, simple pointwise mutations of the structural representatives obtained for the α-variant can thus be assumed to represent viable starting points for MD simulations of the different variants.

#### GEMS

All MD simulations with the GEMS method were performed using the SchNetPack (*75*) MD toolbox with a timestep of 0.5 fs and without any bond constraints. Simulations for polyalanine systems were performed on NVIDIA V100 GPUs on GCP, whereas crambin simulations were performed on NVIDIA A100 GPUs with 80 GB. To mimic experimental conditions (*37*), the simulations of AceAla_15_Lys + H^+^ helix stability were performed in the NVE ensemble starting from an optimized structure with initial velocities drawn from a Maxwell-Boltzmann distribution at twice the simulation temperature as explained above. The folding simulations of AceAla_15_Nme were performed in the NVT ensemble at 300 K starting from the optimized FES using the same method to assign initial velocities. Simulations of crambin in aqueous solution were performed in a simulation box with 8205 explicit water molecules in the NPT ensemble at a temperature of 300 K and a pressure of 1.01325 bar, starting from an optimized structure and initial velocities drawn from a Maxwell-Boltzmann distribution according to the simulation temperature (the first 1 ns of dynamics was discarded to allow the system to equilibrate). Constant temperature and/or pressure simulations use the Nosé-Hoover chain thermostat/barostat (*76*) implemented in SchNetPack using a chain length of 3. Note that simulations in aqueous solution with GEMS use a single MLFF to describe all (solute-solvent and solvent-solvent) interactions in a unified manner.

### Comparison to experimental terahertz spectra of (*48*) (see Fig. 7)

Four MD simulations of crambin in aqueous solution (see above) of 500,000 frames each, sampled every 2.5 fs, were collected. The frames were stripped of water and aligned to calculate a mass-weighted covariance matrix. The eigenvectors of the covariance matrix were taken to indicate resonant vibrations of the molecule according to a quasi-normal mode approximation, and the eigenvalues were used to determine resonant frequencies. An IR spectrum was calculated by calculating a dipole moment for the average structure when modified by each eigenmode displacement. The scale of each displacement was chosen proportional to the inverse frequency. For better correspondence to the experimental IR spectra, which were collected in a partially solvated environment, solvent screening to a cutoff range of 3.3 Å was added for each displaced structure using the 3DRISM liquid state theory (*77*). Inclusion of solvent effects to either greater or lesser range was found to obscure the features of the resulting spectrum. The presented spectra are calculated as a sum of Gaussian peaks with the arbitrary width 4 cm^−1^ and heights assigned as the magnitude of the calculated dipole moment of the fluctuation. Classical MD simulations using the AMBER FF followed the same procedure, except that two longer simulations with 10 million frames each (sampled every 10 ps) were used.

### Simplified model for crambin dynamics

Langevin dynamics for the toy model (see Eq. 1) were computed using the integrator proposed in (*78*) at a temperature of 1 a.u. (arbitrary units) with a timestep of 0.01 a.u. for a total duration of 10 000 a.u. and a friction coefficient γ ≈ 5.13 a.u. (corresponding to 5% stochastic motion). To simulate a “GEMS-like” trajectory, barrier heights and separations were chosen as $h_{i}∼U\left(0.1,5.0\right)$ a.u. and $a_{i}∼U\left(0.1,10.0\right)$ a.u. For the “AmberFF-like” trajectory, the parameters for 90% of the modes were chose as $h_{i}∼U\left(0.1,1.25\right)$ a.u. and $a_{i}∼U\left(0.1,2.5\right)$ a.u., whereas for the remaining 10% of modes, they were chosen as $h_{i}∼U\left(3.75,5.00\right)$ a.u. and $a_{i}∼U\left(7.5,10.0\right)$ a.u. We find that similar results can be obtained for a range of parameter values, as long as there is a clear separation of large conformational changes with large energy barriers and small conformational changes with small energy barriers (to produce AmberFF-like trajectories), or no such separation (to produce GEMS-like trajectories).

## Supplementary Materials

### This PDF file includes:

Sections S1 to S6 Figs. S1 to S26 Legends for movies S1 and S2 References
