# Supplementary material for: Biomolecular dynamics with machine-learned quantum-mechanical force fields trained on diverse chemical fragments
Source: Sci Adv. 2024 Apr 5;10(14):eadn4397. doi: 10.1126/sciadv.adn4397 (PMC11809612; doi:10.1126/sciadv.adn4397)
Supplement: Supplementary file 1 — Sections S1 to S6 Figs. S1 to S26 Legends for movies S1 and S2 References [file sciadv.adn4397_sm.pdf]

Science Advances
AAAS

Supplementary Materials for

Biomolecular dynamics with machine-learned quantum-mechanical force fields trained on diverse chemical fragments

Oliver T. Unke *et al.*

Corresponding author: Alexandre Tkatchenko, alexandre.tkatchenko@uni.lu;
Klaus-Robert Müller, klaus-robert.mueller@tu-berlin.de

Sci. Adv. **10**, eadn4397 (2024)
DOI: 10.1126/sciadv.adn4397

The PDF file includes:

Sections S1 to S6
Figs. S1 to S26
Legends for movies S1 and S2
References

Other Supplementary Materials for this manuscript includes the following:

Movies S1 and S2

S1 Background

Conventional force fields (FFs) allow to study large systems, e.g. entire viruses (*81–83*), in atomic detail. They achieve this remarkable efficiency by modeling chemical interactions as a sum over simple empirical terms (*84–86*). However, while very efficient, their accuracy is limited (*87*) and they typically cannot describe chemical reactions. Although there are various efforts to increase the accuracy of classical FFs, for example by including polarization effects (*88, 89*) and sophisticated models for anisotropic charge distributions (*90–94*), or by developing reactive FFs (*95, 96*), they are clearly much faster to evaluate but typically cannot compete with the accuracy of machine learned force fields (MLFFs) (*97–103*). Machine learning (ML) methods "learn the rules" of quantum mechanics (*104*) and their representation from data, allowing to skip computationally expensive *ab initio* simulations. Beyond FF construction, there are several other applications of ML to quantum chemistry (QC). One of the earliest uses of ML in QC was the exploration of chemical space (*105–108*). However, ML can also be used to accelerate studies that typically rely on MD simulations or other dynamical equations (*109*). For example, it can be used to directly sample equilibrium distributions (*110, 111*) or rare events (*112*), or directly predict reaction rates (*113*). Further, ML is used for predicting protein structure (*114–116*), solving the Schrödinger (*117–119*), predicting wave functions (*120–122*), modelling solvated systems (*123*), generating molecules and solving inverse design problems (*124–130*), and even for planning chemical syntheses (*131*).

For a more detailed overview of the use of ML in molecular and material science, refer to Refs. 132–137, for an overview of applications in molecular simulations, refer to Ref. 138, and for reviews on the exploration of chemical space, refer to Refs. 139 and 140, furthermore general reviews can be found in Refs. 104, 131, 141–146.

S2 MD simulations with conventional force fields

S2.1 Equilibration and detailed setup

After initial preparation (resolving doubly- or ill-defined residues and atom type definitions present in the original files from the respective sources specified in the main manuscript), classical molecular dynamics (MD) simulations of solvated systems were initialized by resolvating the systems in cubic simulation boxes with a minimum protein-to-box distance of 1.6 nm. Unless explicitly specified otherwise, simulation cells were solvated in TIP3P water with physiological concentrations of NaCl with excess Na^+- or Cl^--ions to neutralize the simulation box where needed. The solvated structures were subsequently optimized to a maximum atomic force of 1'000 kJ/mol/nm and equilibrated in a four-step procedure consisting of (a time step of 2 fs was used in all cases):

1) a short NVT-simulation of 50'000 steps (simulation time 100 ps)

2) NPT simulation (Berendsen barostat) of 50'000 steps (100 ps)

3) NPT simulation (Parrinello-Rahman barostat (*72*)) of 100'000 steps (200 ps) with fully constrained bonds

4) and 100'000 steps (200 ps) with constraints on all bonds involving hydrogen.

In all equilibration runs a constant temperature thermostat with stochastic velocity rescaling (*71*) set to the final simulation temperature was employed. Throughout all steps, the AMBER99SB-ILDN force field (*24*) was used.

For $AcAla_{15}Lys$-chains involving the charged $LysH^+$ terminus, topology and AMBER definitions have been adapted accordingly using the default AMBER99SB-ILDN parametrization.

For the gasphase $AcAla_{15}Lys{+}H^+$, we adopted pseudo-gasphase settings as detailed in Ref. 70 using maximal unit cells while disabling particle-mesh Ewald electrostatics. The

constant temperature (pseudo-)gasphase simulations were prepared by structure optimization (maximum atomic force of 1'000 kJ/mol/nm). The reported simulations were then run in an NVT ensemble initialized with velocities randomly drawn from a Maxwell-Boltzmann distribution correponding to twice the simulation temperature.

S3 Sampling structures for top-down fragmentation

To train a model that can be used to simulate trajectories of a particular system of interest, we want to train it on a diverse set of top-down fragments representative of a variety of conformations. The general strategy is to cluster configurations that occur in classical MD simulations, select some representatives for each cluster, and then decompose the whole configurations into spherical regions that are small enough to run DFT calculations on them (top-down fragments). Different systems have different characteristics when it comes to the possible conformations:

- The poly-alanine systems unfold and thus show a lot of variation, but the overall system is comparatively small (contains few atoms).
- Crambin in aqueous solution contains many different atoms, but due to presence of three disulfide bridges, the protein itself shows variations mainly determined by the states of the disulfide bridges, so clustering is straightforward.

Poly-alanine systems

In our classical MD simulation of $\mathrm{AceAla_{15}Nme}$ and $\mathrm{AceAla}_n\mathrm{Lys} + \mathrm{H}^+$ in solution at temperatures 280K, 300K, and 310K, (2 μs each) the poly-alanine chain did not keep a helix structure, but assumed almost arbitrary conformations. So we cannot define different well defined clusters, but we can still use a clustering algorithm to find a diverse and representative sample of the configurations seen during the trajectories.

We used affinity propagation (*147*) as the clustering algorithm. This algorithm takes as input a matrix specifying "similarities" between two objects, and a "preference" that specifies the cost of adding a new cluster (which is balanced with the gain in similarity obtained by switching nearby objects to the new cluster). The output is a set of clusters with one object in each cluster designated as the representative of this cluster. The number of clusters is controlled by the relation between similarities and the preference; default choices for the preference include the median similarity and the lowest similarity, but in general the preference can be tuned to produce clusters at the desired granularity.

To compare two configurations of atoms, we move them so that the center of mass is at the origin, and then use the rotation that gives the minimal mean square distance between the atoms. The similarity is then the negative sum of the square distances. We set the preference to -50 compared to a median similarity between -14 and -31 for the six trajectories, this gave 240 cluster for AceAla$_n$Lys + H^+ molecule, and 266 cluster for the AceAla$_{15}$Nme molecule.

Crambin

Initial structure were taken from PDB entry 2FD7. The incorrect residues SER11 and VAL15 have been remodeled using PyMOL.

Crambin has 3 disulfide bridges at atoms (NCCS:SCCN)

- 31-33-35-38:561-558-556-554
- 41-43-45-48:449-446-444-442
- 220-222-224-227:373-370-368-366

These disulfide bridges have two stable positions, in two MD simulations over 5 µs each we observed the first and the third disulfide bridge to flip between stable positions (measured as dihedral angle of the N-C-C-S configurations). Together with a twist at an Arginine residue, these

explained almost all the variations seen. We computed 22 clusters and made sure all observed variations were represented.

S4 Comparison to ground truth for AcAla$_{15}$NME trajectories

To check the accuracy of our GEMS simulation, we select samples from 100 trajectories of 2500 steps starting from a common stretched initial conformation. We subsample by only taking every second time step, which leaves 125,000 conformations. We use affinity propagation (see Section S3) to get representative samples. The similarity is the negative sum of square distances between corresponding non-hydrogen atoms, after centering the molecules and applying an optimal rotation.

However, using affinity propagation directly on these trajectories would have a large bias towards stable end conformations: Our trajectories contain stable end conformations for roughly half of the time, so affinity propagation with the default settings would spend most representatives on the stable conformations, largely ignoring the interesting folding part. To reduce this bias, we use a preprocessing step that removes conformations that have a small distance to an already selected point. (The threshold used was 9 Å^2 for the sum of the square distances, corresponding to 0.3 Å per non-hydrogen atom for the RMSE.)

This reduces the stable tails of the trajectories, but if we do this only within the trajectories, we get another bias around the common initial conformation. Computing pairwise distances for the union of all trajectories would be computationally expensive, so we use an approximation: We randomly mix all 100 trajectories and subdivide them into a partition of smaller subsets, and remove “almost duplicates” (as above) only inside each of the partitions. We then mix again and thin out again three more times. This removes most of the “almost duplicates”, and we arrive at 25,249 conformations from the original 125,000 conformations. On these 25,249 we can then

run the affinity propagation; using a preference of -1500 Å^2 we arrive at 1554 representatives. Plotting the distance from the nearest representative (blue curve) over the trajectories now shows an even average distance: The red curve is a rolling average over 100 time points, it hovers around 0.4 Å. The x-axis gives the time steps in the simulation. After every 2500 steps = 250 ps the next trajectory starts, so in the image below there are data from the first two trajectories. In the blue curve, conformations that are selected as cluster representatives can be seen as time points in which the blue curve touches the x-axis (since the distance to the closest representative is then 0). We can see that there are regions that need more representatives (e.g. when folding happens), and regions which only have occasional representatives (in the more stable end phase), but the average distance stays approximately constant (see Fig. S1). While this takes care of any

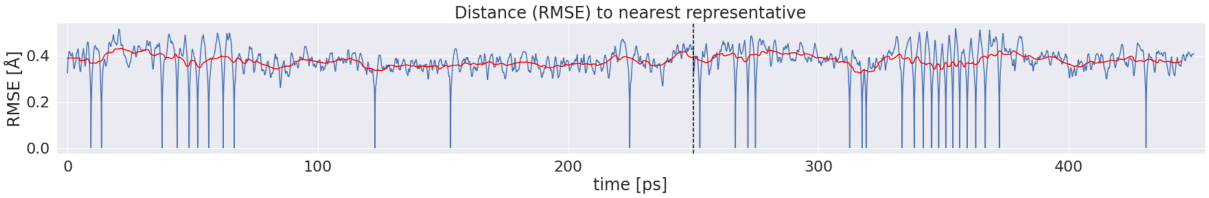

Figure S1: **Distance (RMSE) to nearest representative for AcAla$_{15}$NME trajectories.** The blue curve shows the instantaneous distance, while the red curve shows the average.

obvious bias towards common or stable positions, we also add a list of 1000 conformations that are as far away as possible from all previously selected conformations. These can be thought of as untypical or unstable conformations, and we want to make sure that our model works for them as well as it does for the maybe more typical cluster representatives.

S5 MD simulations with GEMS

The MD simulations with GEMS are performed with the SchNetPack (*77*) toolbox providing an interface to the Atomic Simulation Environment (*148*) to run MD simulations with machine

learning models. SchNetPack includes a fully functional MD suite, which can be used to perform efficient MD and PIMD simulations in different ensembles. The SpookyNet (*17*) model is used to implement

```
schnetpack.md.calculators.MDCalculator
```

interface from SchNetPack. See figure S2 for the schematic and corresponding papers for more details. Both SpookyNet and SchNetPack are written in PyTorch and thus can be used to run

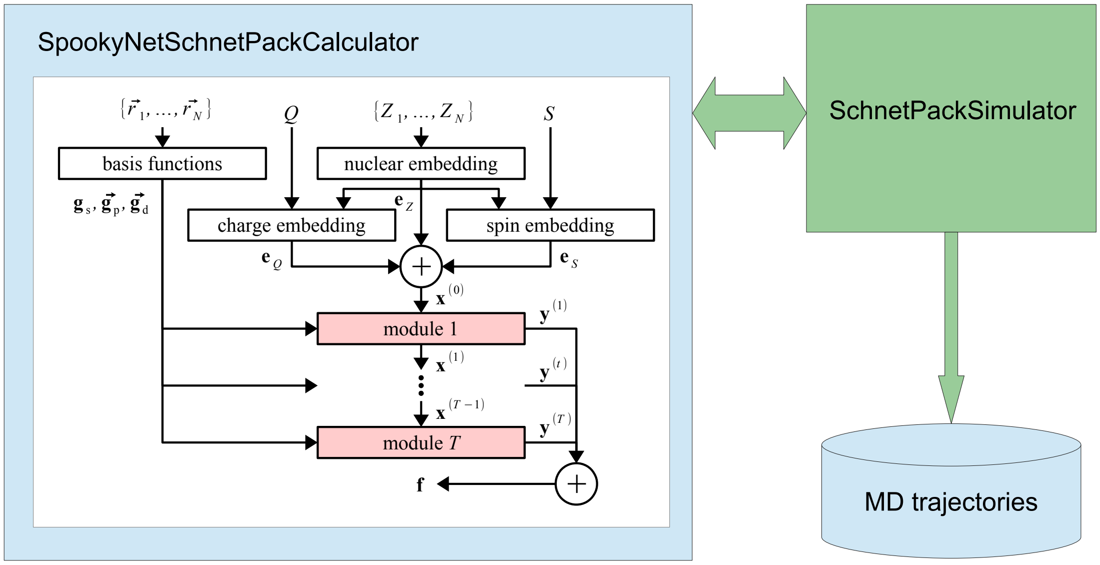

Figure S2: **MD implementation**. Only the blue box (schnetpack.MDCalculator subclass using SpookyNet model to get forces predictions from atom positions and charges) needs to be implemented. SchNetPack Simulator takes care of running MD simulation, checkpointing and writing logs and trajectories to disk.

S6 Transferability of GEMS models trained on different top-down fragments

Since top-down fragments are system-specific, it is instructive to investigate the transferability of GEMS models to systems that are not covered by the top-down fragments they were trained on. To this end, we apply the model for crambin to the prediction of the gas-phase ACE2-RBD binding curves shown in Fig. 9 and the folding of $\text{AceAla}_{15}\text{Nme}$. We find that compared to the system-specific GEMS model, ACE2-RBD binding energies are systematically over-predicted, but relative binding strengths of SARS-CoV-1 and SARS-CoV-2 match closely (see Fig. S4). For the folding of $\text{AceAla}_{15}\text{Nme}$, we find that trajectories with the crambin model follow the same folding mechanism (via "wavy intermediate") observed for the system-specific model. However, the formed helix seems to be more biased towards an α-helical conformation with less 3_{10}-helical content (see Fig. S5). To be specific, while a 10 ns trajectory of the helical state with the poly-alanine-specific GEMS model suggests a $\sim$38/62 mixture of α- and 3_{10}-helices, the crambin-specific GEMS model predicts roughly 80%–90% α-helical content in the helical state. This suggests that while GEMS models are somewhat transferable even without system-specific training, quantitative results seem to require system-specific top-down reference data. However, it might still be possible to construct top-down reference data covering a large class of proteins, such that a single GEMS model is able to describe multiple systems with the same accuracy as a system-specific model.

As an additional extreme test of transferability, we apply the crambin-specific GEMS model and a variant trained only on bottom-up fragments, to a 5 ns NPT simulation of a pure water box containing 8393 water molecules at 300 K and ambient pressure. Note that the solvated protein fragments dataset (*67*) we use as basis for the bottom-up fragments contains almost exclusively pure or (micro-)solvated protein fragments. Less than 0.4% of the dataset correspond to structures

containing only water molecules, with at most 40 water molecules in total (see Ref. 19 for details). While top-down fragments constructed from the protein-solvent interface contain some water molecules, by construction, the top-down fragments never contain only water molecules. As such, both GEMS models investigated here are strongly biased towards the correct description of the protein-water interface and should not be expected to give quantitative results for bulk water. Nonetheless, we find that both variants of GEMS predict the density of water reasonably well as 1014±2 kg/m^3 (crambin model) and 955±2 kg/m^3 (general fragments only) compared to the experimental density of 996 kg/m^3. The oxygen-oxygen radial distribution functions (see Fig. S3) predicted by GEMS indicate that overall, the water is "too structured" compared to the experiment. This effect has also been observed for Car-Parrinello MD simulations of liquid water (*149*), where it was found that the inclusion of nuclear quantum effects (NQEs) can significantly improve the agreement between simulations and experiments. Similarly, simulations at the PBE0+TS-vdW(SC) level of theory also found the water to be "too structured" (*37*), and approximately incorporating nuclear quantum effects by raising the simulation temperature to 330 K was found to significantly improve agreement with experiment. A similar effect can be seen for GEMS simulations at 330 K, where the increased temperature leads to a better agreement with the experimental reference (see Fig. S3). Another possible explanation for the discrepancies is that due to the training data, GEMS is biased towards the description of water at protein interfaces, where it is generally more structured than in bulk water.

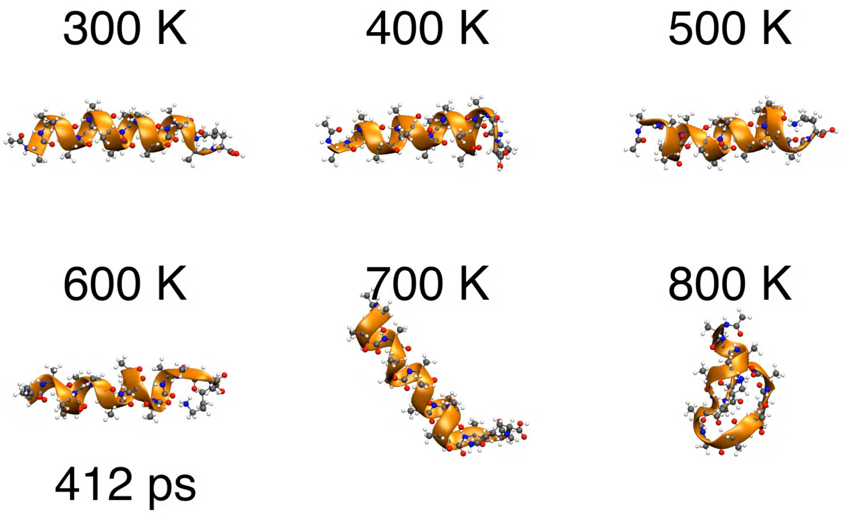

Video S1: **Thermal stability of AceAla$_{15}$Lys + H^{+}**. Full video available at `https://youtu.be/QZIc3a4OjJk`

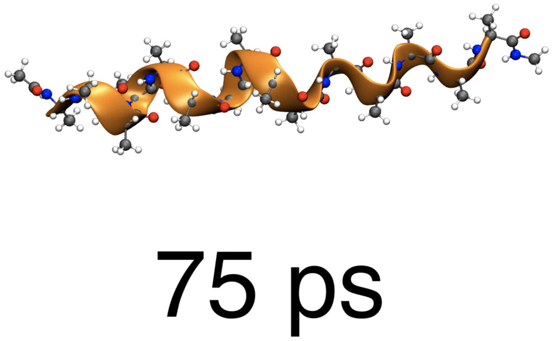

Video S2: **Folding of AceAla$_{15}$Nme**. Full video available at `https://youtu.be/ZuKW292DKKw`.

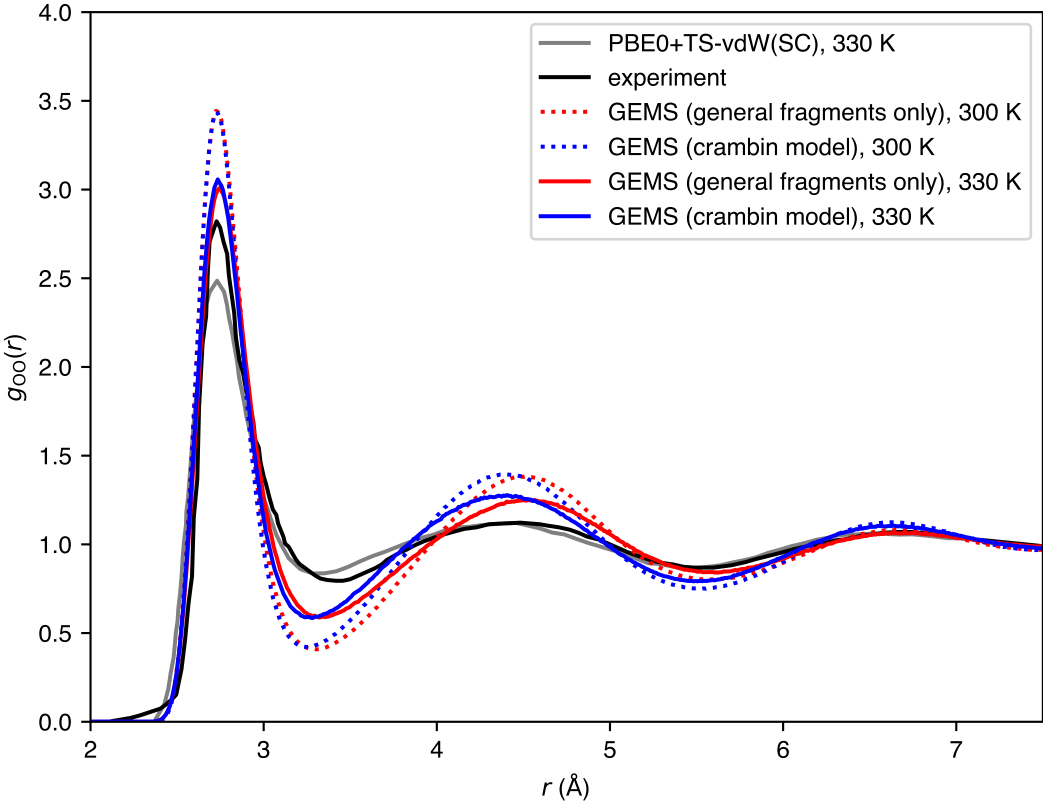

Figure S3: **Oxygen-oxygen radial distribution function for 5 ns long GEMS simulations of pure bulk water (8393 water molecules)**. Experimental results are taken from Ref. 150, results for PBE0+TS-vdW(SC) from Ref. 37.

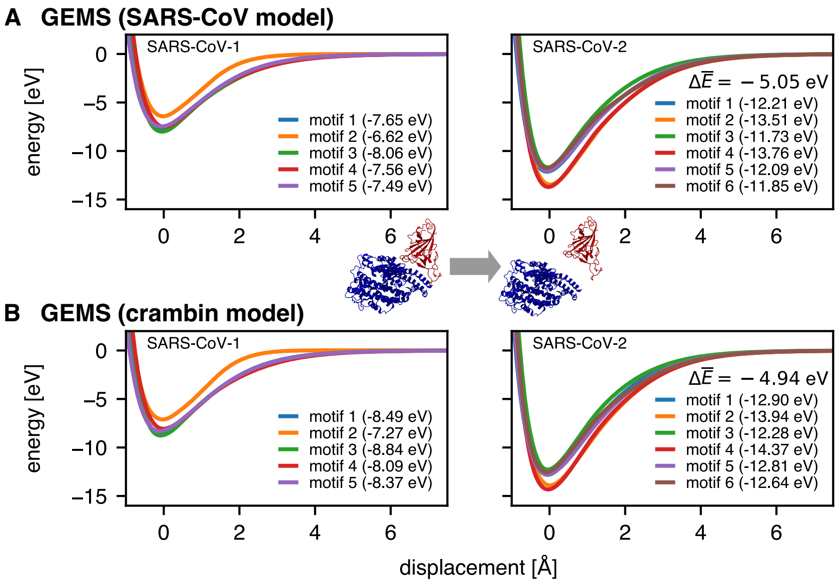

Figure S4: **Gas-phase binding curves of the ACE2 (blue) and the receptor binding domain (RBD) of the SARS-CoV spike protein (red) predicted with the system-specific GEMS model in comparison to the crambin model**. While the crambin model systematically predicts stronger binding, the average difference in well-depth $\Delta\overline{E}$ between SARS-CoV-2 and SARS-CoV-1 closely matches the value predicted by the system-specific value.

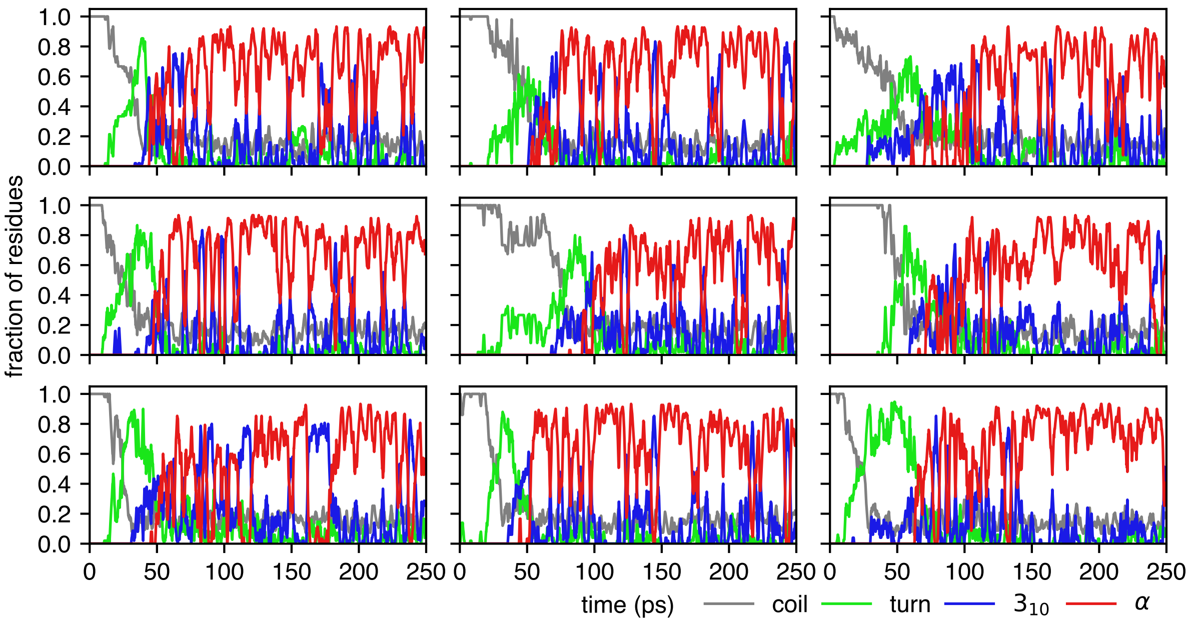

Figure S5: **Secondary structural motifs (determined by STRIDE (*38*)) along typical folding trajectories of AcAla$_{15}$NME simulated with the GEMS model trained for crambin.** Starting from a random coil, AcAla$_{15}$NME quickly folds into a helical state via an intermediate that is primarily classified as turn.

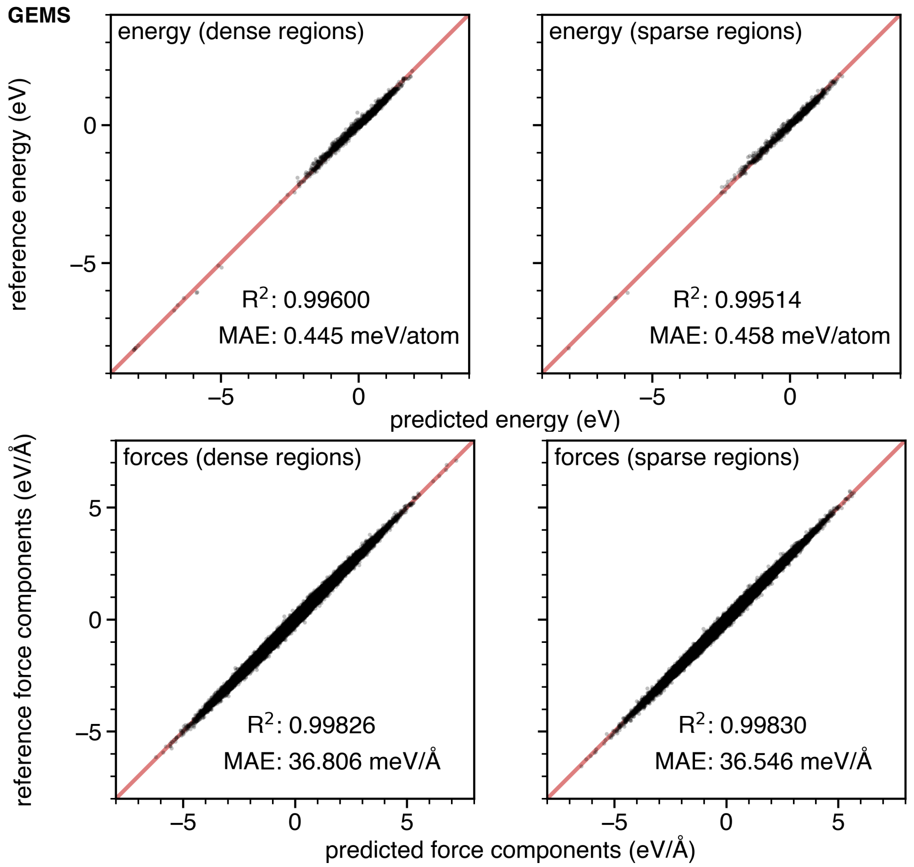

Figure S6: **Correlation of predicted and *ab initio* reference (ground truth) energies and forces for AceAla$_{15}$Nme conformations sampled from 100 aggregated 250 ps MD trajectories (25 ns total) in the NVT ensemble at 300 K simulated with GEMS.** Conformations are sampled either from densely (1554 structures) or sparsely (1000 structures) populated regions of conformational space.

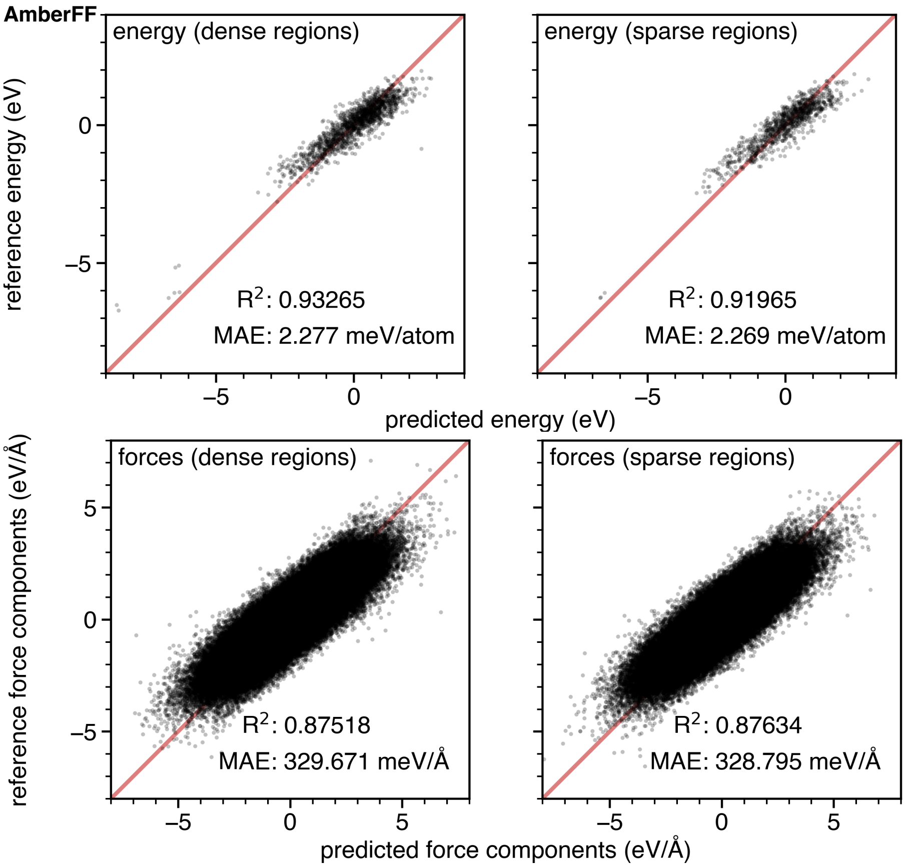

Figure S7: **First alternative correlation plot of *ab initio* reference (ground truth) energies and forces.** Same as Fig. S6, but showing the correlation for predictions with the AmberFF.

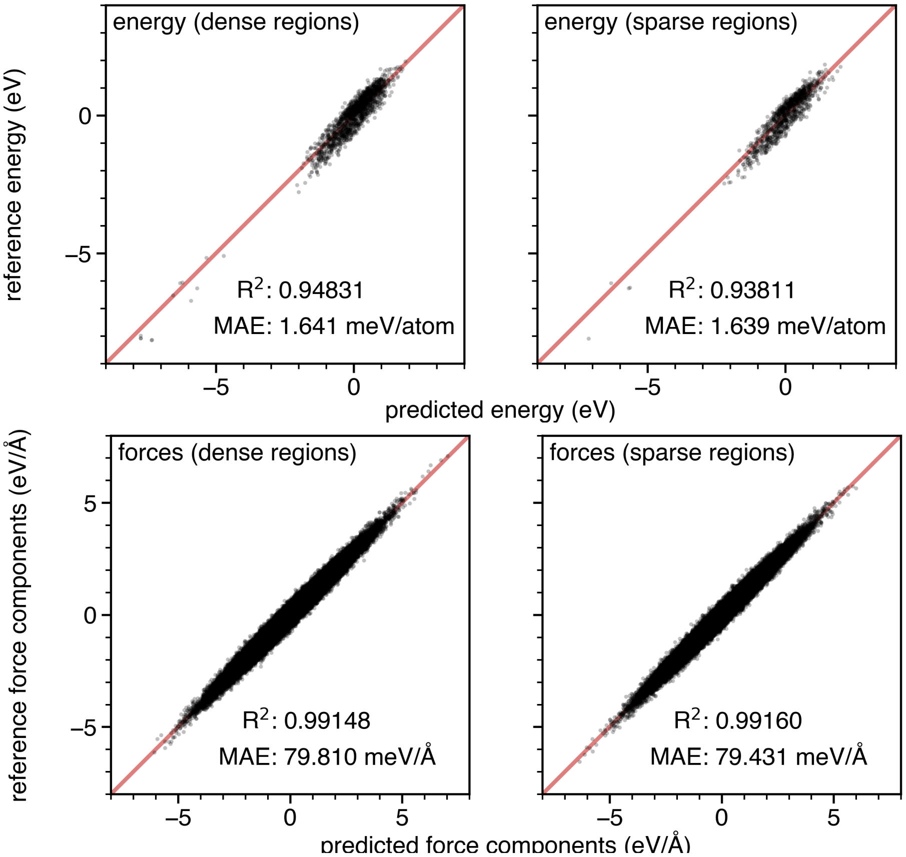

Figure S8: **Second alternative correlation plot of *ab initio* reference (ground truth) energies and forces.** Same as Fig. S6, but for a GEMS model trained without top-down fragments.

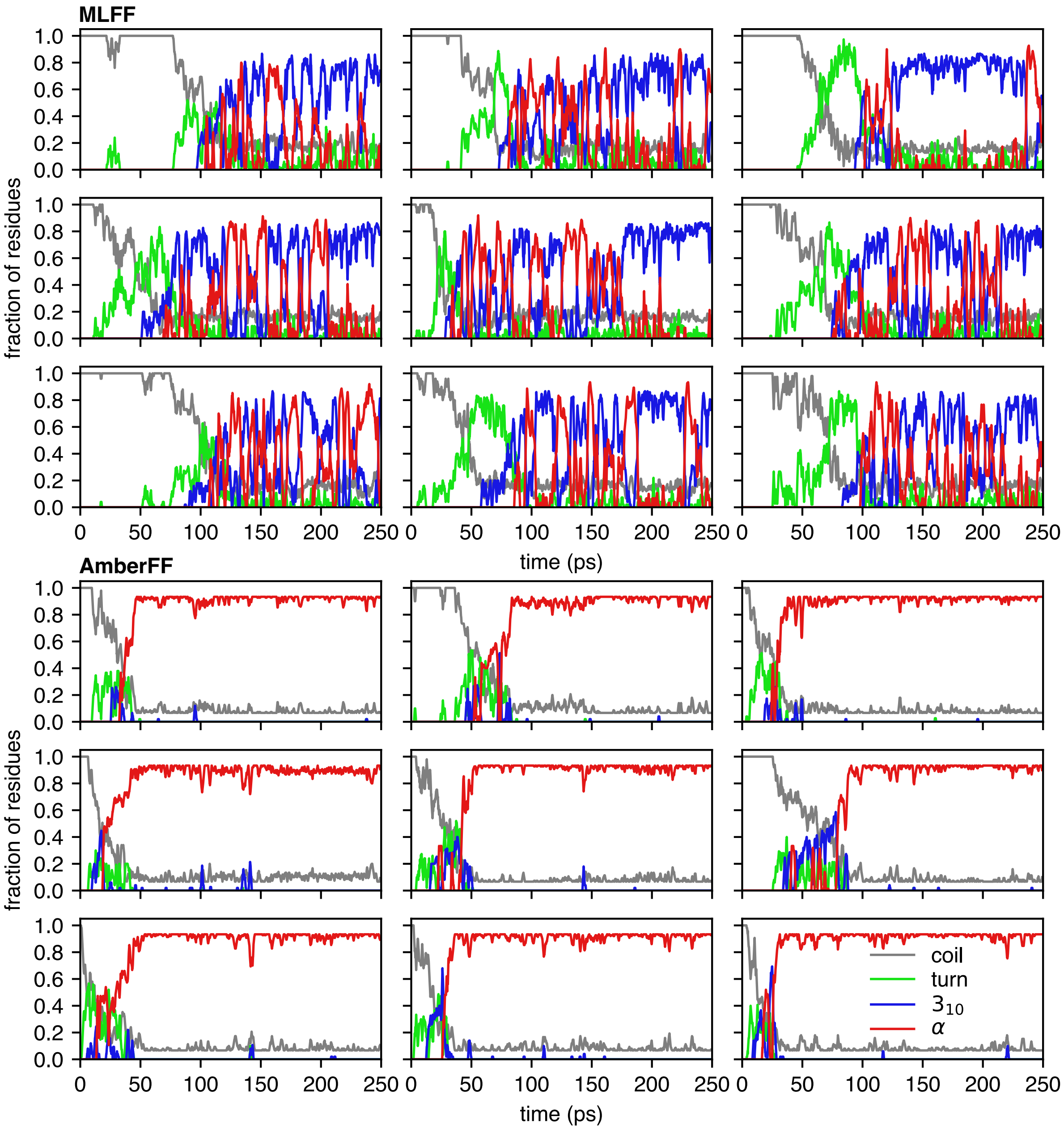

Figure S9: **Secondary structural motifs (determined by STRIDE (*38*)) along additional folding trajectories of AcAla$_{15}$NME.** Starting from a random coil, AcAla$_{15}$NME quickly folds into a helical state. For GEMS, this occurs via an intermediate that is primarily classified as turn and the helical state is a dynamic mixture between 3_{10}- and α-helices, whereas for AmberFF, the peptide directly folds into a rigid α-helix.

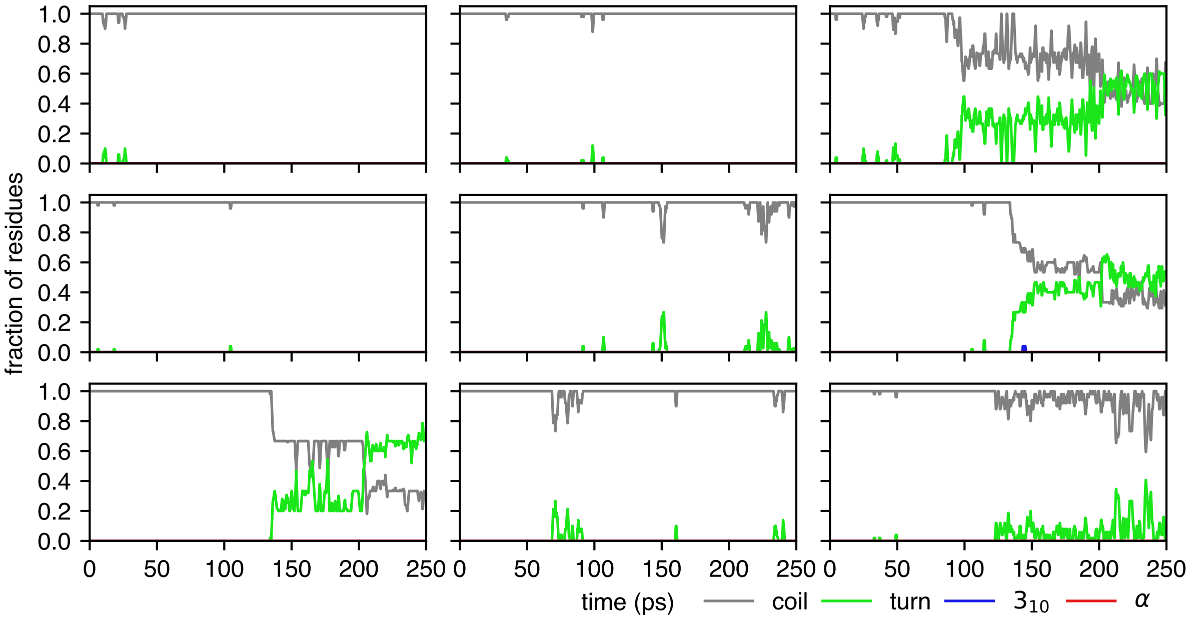

Figure S10: **Secondary structural motifs (determined by STRIDE (*38*)) along representative trajectories of AcAla$_{15}$NME simulated with GEMS* (trained only on bottom-up, but not top-down fragments).** Without learning the correct long-range interactions from the top-down fragments, the model is unable to predict the correct folding process and the peptide primarily stays a random coil.

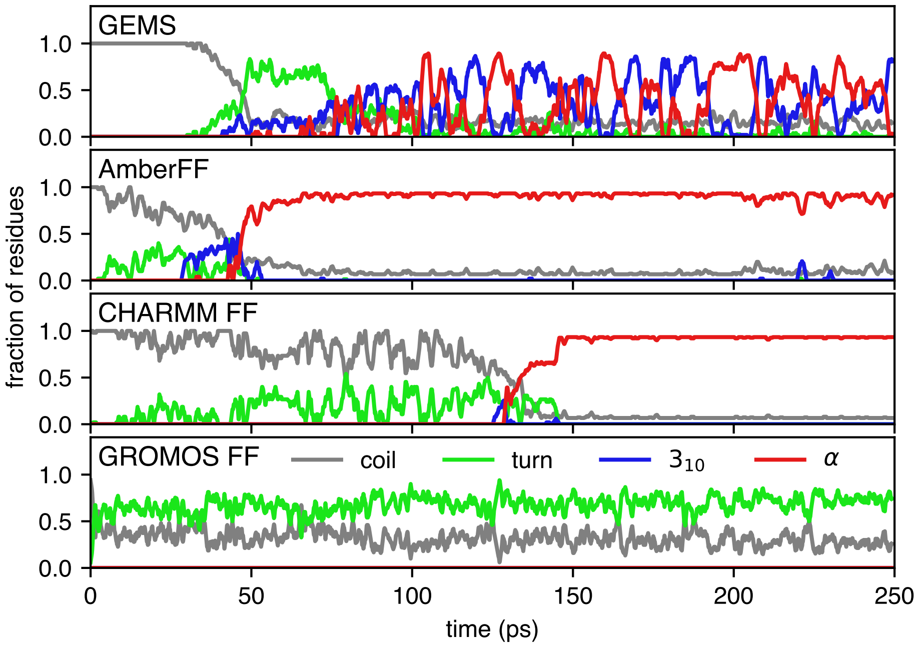

Figure S11: **Secondary structural motifs (determined by STRIDE (*38*)) along representative folding trajectories of AcAla$_{15}$NME simulated with GEMS in comparison to simulations with conventional force fields Amber ff99SB (AmberFF) (*24*), CHARMM27 (CHARMM FF) (*45*), and GROMOS96 53A5 (GROMOS FF) (*46*).** While AmberFF and CHARMM FF both predict folding to a rigid α-helix, GEMS predicts a dynamical equilibrium between 3_{10}- and α-helices.

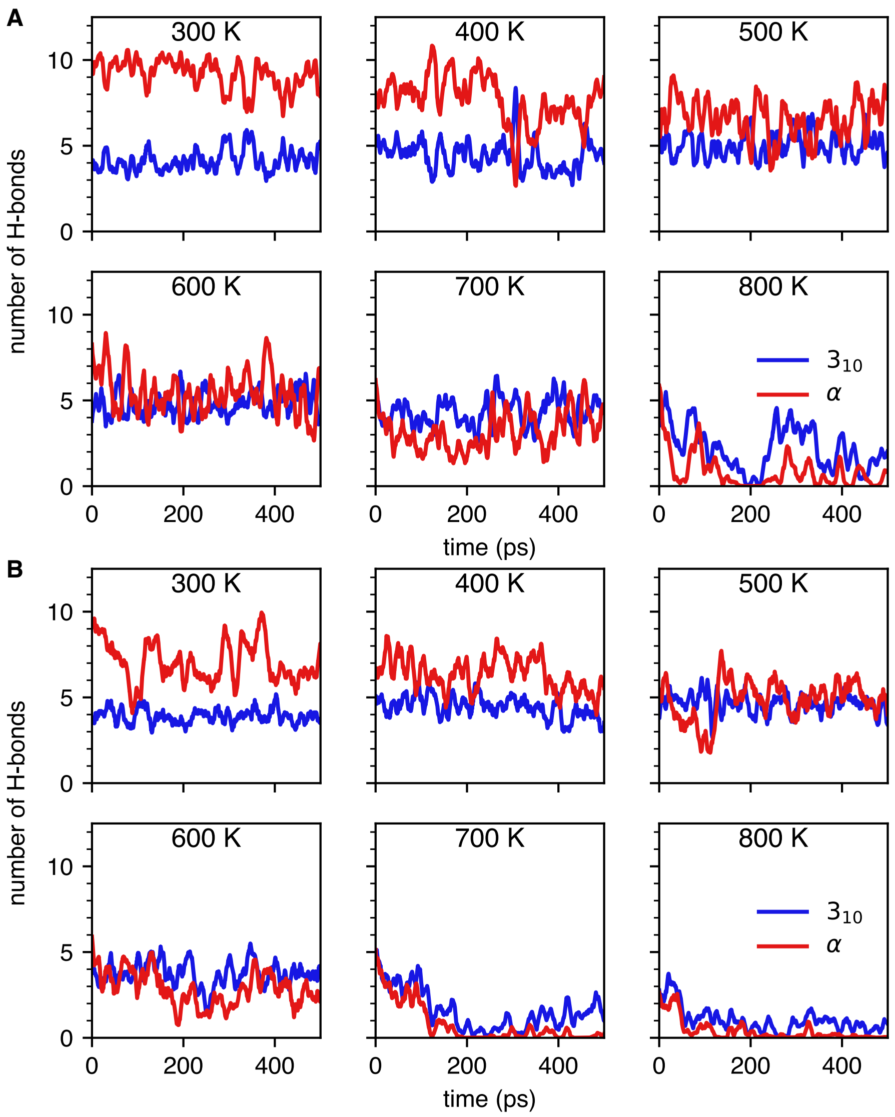

Figure S12: **Number of α- and 3_{10}-helical H-bonds during GEMS MD simulations of helical AceAla$_{15}$Lys + H$^+$ in gas phase at different temperatures.** (**A**) GEMS model trained with top-down fragments. The sharp drop in the number of H-bonds in the dynamics at 800 K indicates the formation of a random coil. (**B**) Same as panel A, but for a model trained without top-down fragments. The number of H-bonds is lower on average for all temperatures and a random coil is formed at a lower temperature.

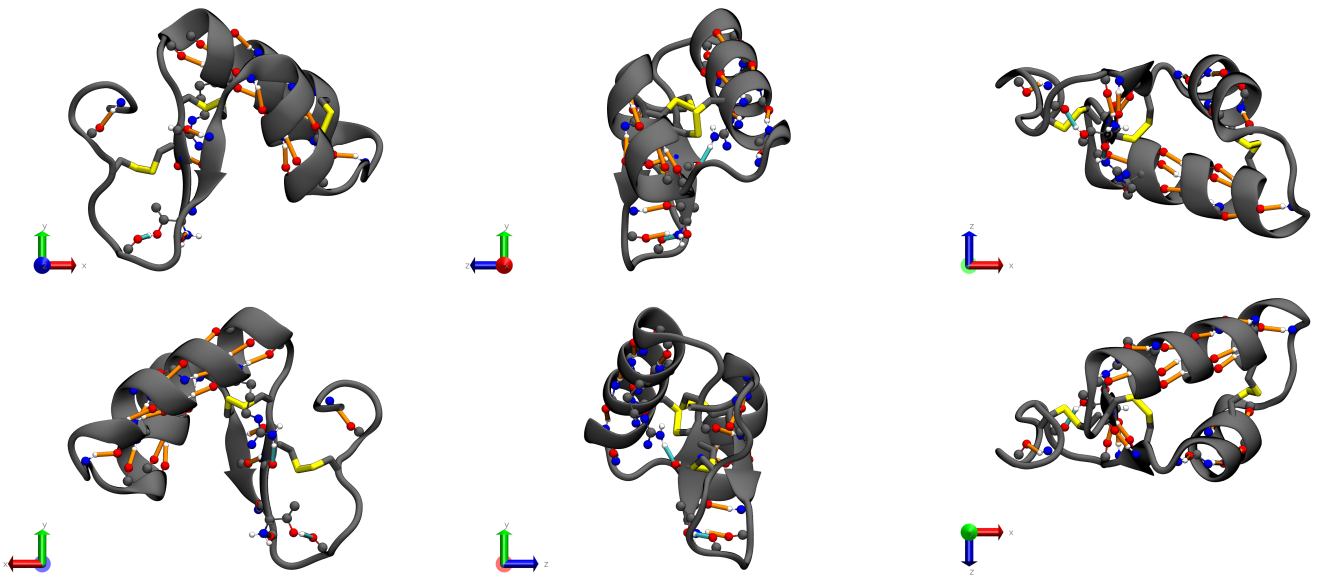

Figure S13: **Covalent and non-covalent interactions in crambin responsible for its three-dimensional structure.** Hydrogen bonds between backbone-backbone and backbone-sidechain atoms are shown in orange and cyan, and disulfide bridges in yellow. Backbone and sidechain atoms are only shown if relevant to one of the interactions. Six different viewpoints are shown.

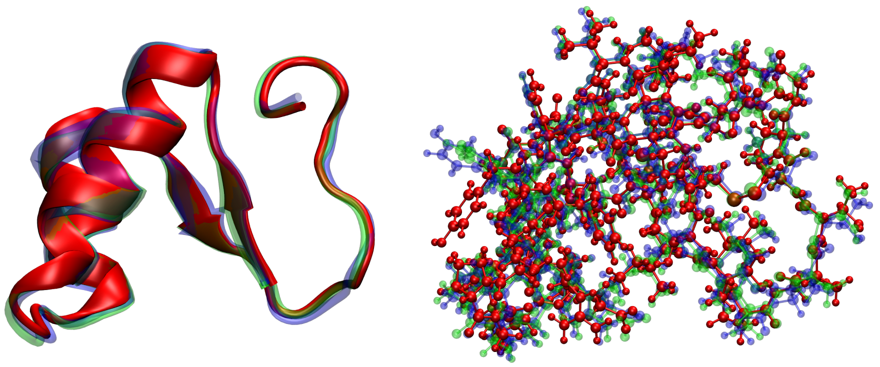

Figure S14: **Structure of crambin (left cartoon, right all-atom) after solvation and subsequent minimization with GROMACS starting from different experimental structures of crambin reported in the Protein Data Bank (PDB).** The results from PDB entries 2FD7 (*48*) (red, resolution 1.75 Å), 1EJG (*75*) (green, resolution 0.54 Å), and 3NIR (*76*) (blue, resolution 0.48 Å) have root mean square deviations (RMSDs) of 0.808 Å, 0.540 Å, and 0.486 Å from the averaged structure, respectively.

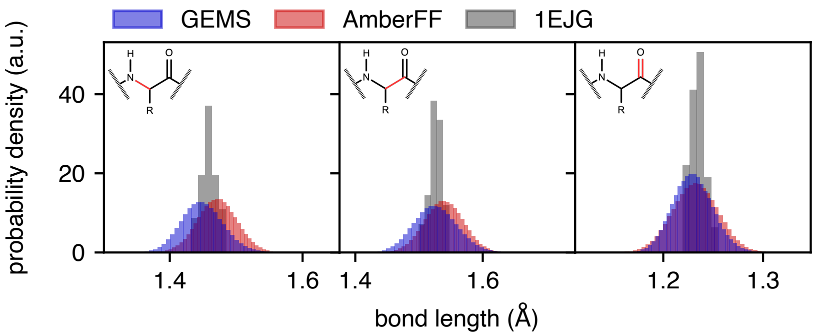

Figure S15: **Backbone bond length distributions in GEMS and AmberFF simulations of crambin compared to a high-resolution crystal structure.** (*75*) GEMS shows systematically shorter bond lengths than AmberFF, but both distributions are consistent with the experimental reference.

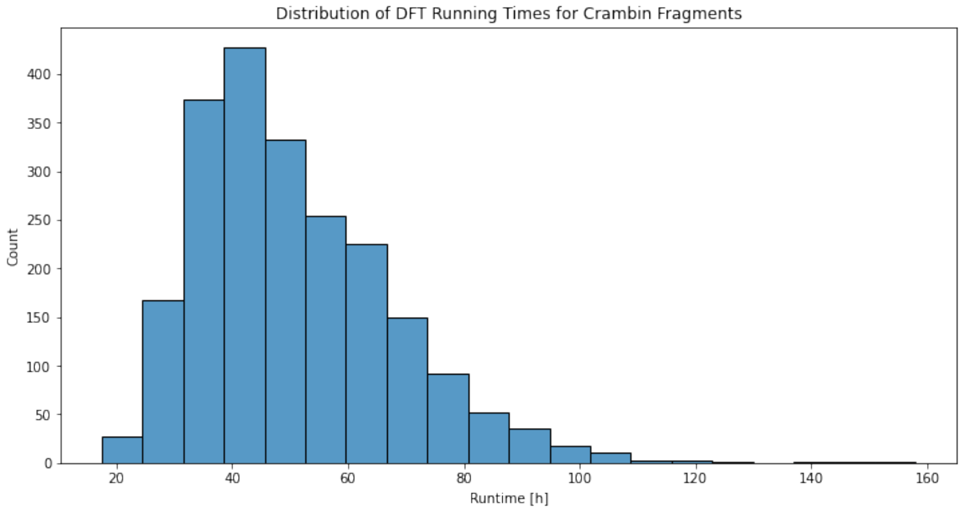

Figure S16: **DFT runtime distribution for crambin fragments.** The majority of calculations finish after around two days.

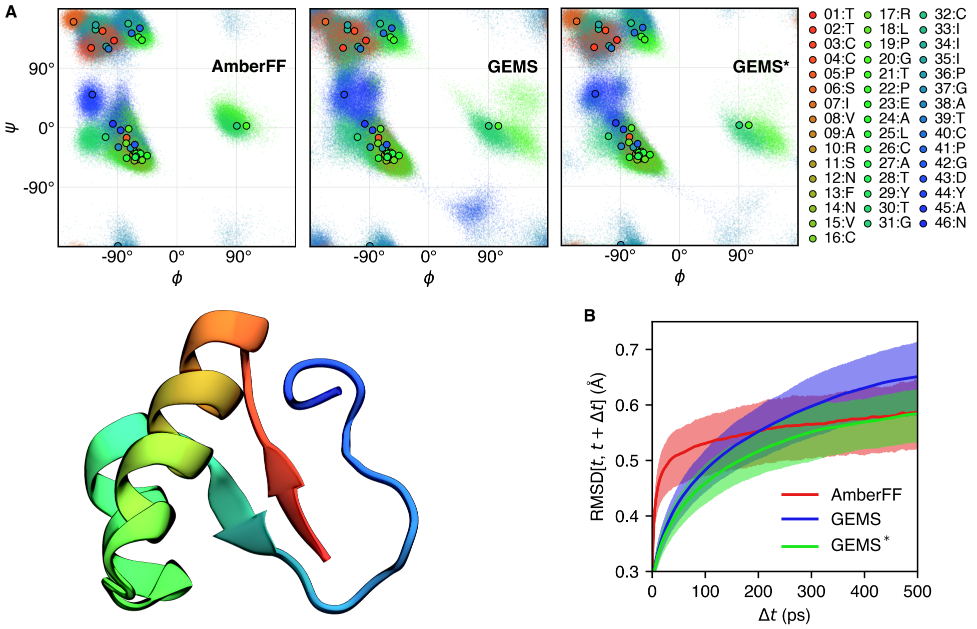

Figure S17: **Effect of top-down fragments on crambin dynamics. A GEMS model trained only on bottom-up fragments and without any top-down fragments (GEMS*) is compared to the results of AmberFF and a regular GEMS model.** (**A**) Ramachandran map for crambin (color-coded by residue number) (see also Fig. 4B). Although the results for GEMS* are qualitatively similar to those of GEMS, some regions in the Ramachandran map are sampled less frequently and appear closer to the results observed for AmberFF. (**B**) Distribution of root mean square deviations (RMSDs, excluding hydrogen atoms) between conformations sampled at times t and $t + \Delta t$ (see also Fig. 4D). Dynamics with the GEMS* (green) resembles those of GEMS (blue) for small values of Δt, but fluctuations for large Δt are closer to those of AmberFF.

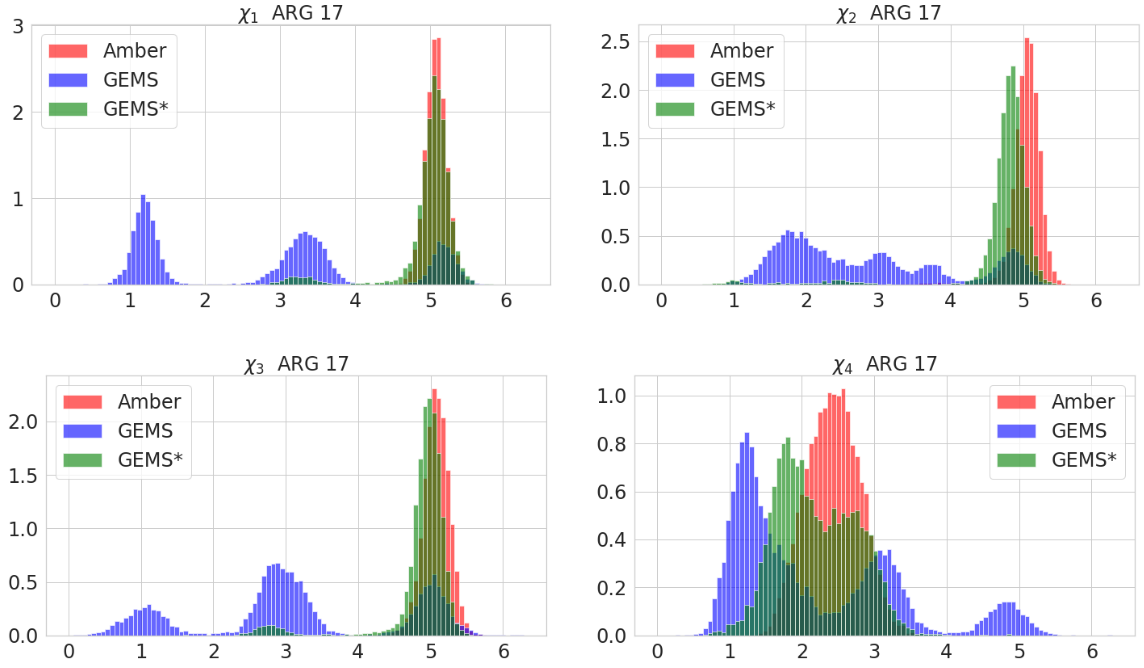

Figure S18: **Torsion angles for residue 17, arginine.** x–axis: Angle, y–axis: Density. The longest residues, Arginine, give (together with the disulfide bridges) the largest contributions to systematic differences in the conformation. The structure of the Arginine residue is given by its 4 torsion angles, their distribution is plotted with respect to Amber, GEMS, and GEMS* trajectories. GEMS shows more flexibility than Amber, GEMS* is somewhere in between, but closer to Amber.

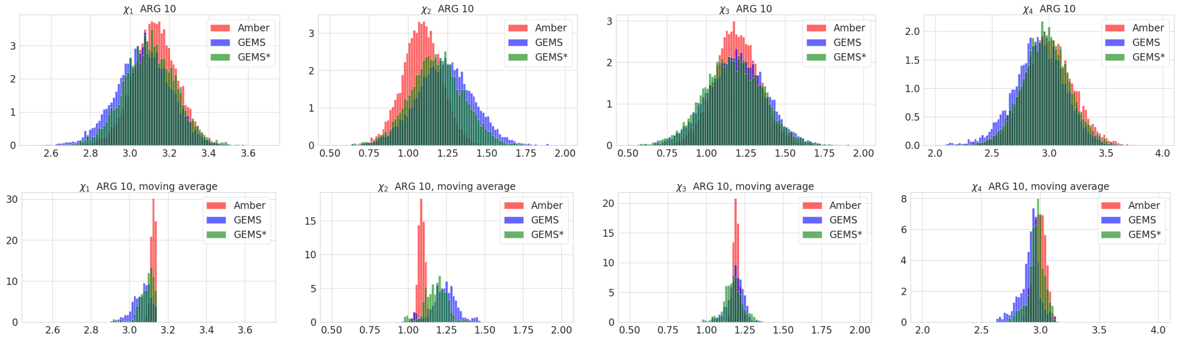

Figure S19: **Torsion angles for residue 10, arginine, top: original, below: averaged over 100 time steps.** Amber oscillates more on fast timescales, but around a well defined ground state. GEMS has more variations of the ground state for χ_1, χ_2, χ_3.(It seems χ_4 is an exception in which GEMS behaves like Amber.)

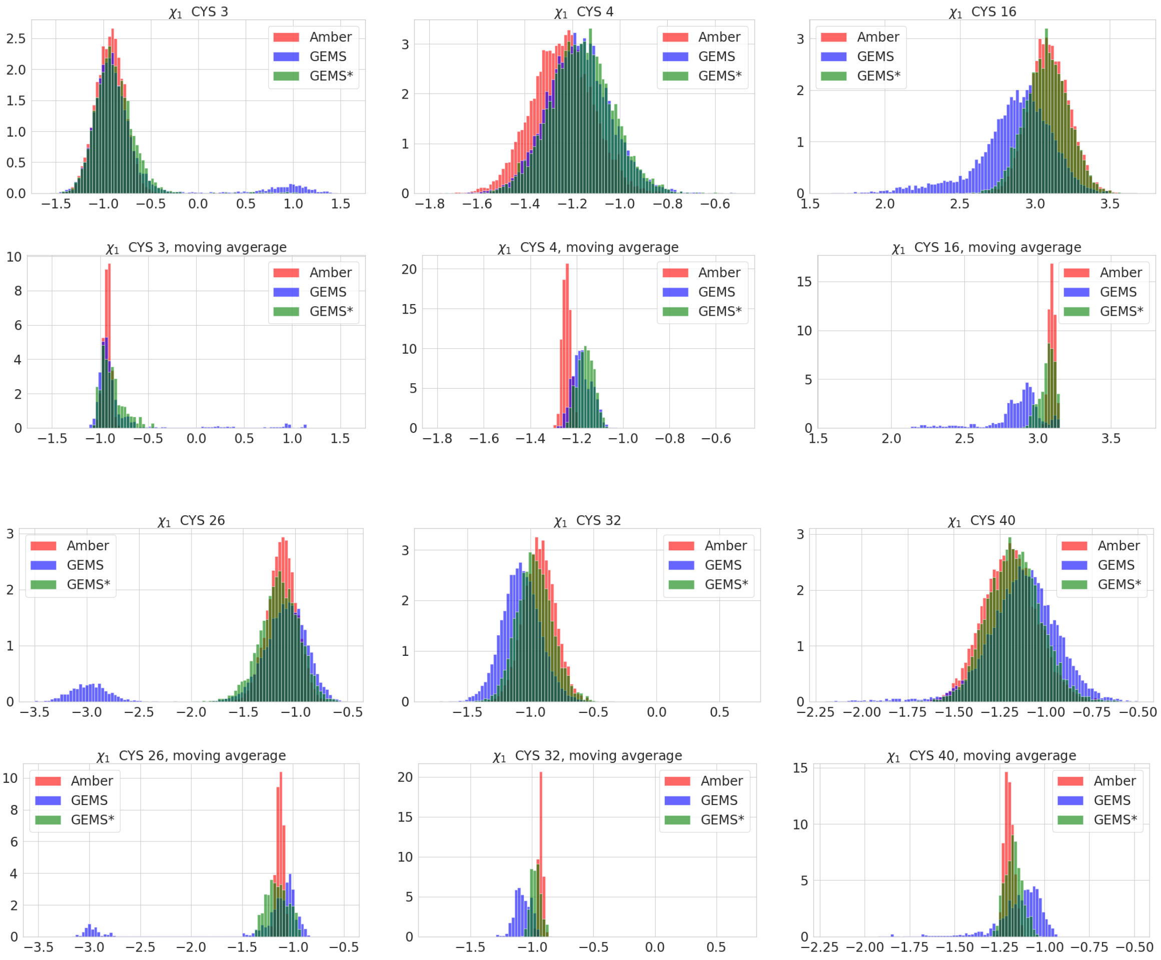

Figure S20: **Torsion angles for cysteine residues. Rows 1 and 3: original, rows 2 and 4: averaged over 100 time steps.** Amber oscillates more on fast timescales, but around a well defined ground state. GEMS has more variations of the ground state.

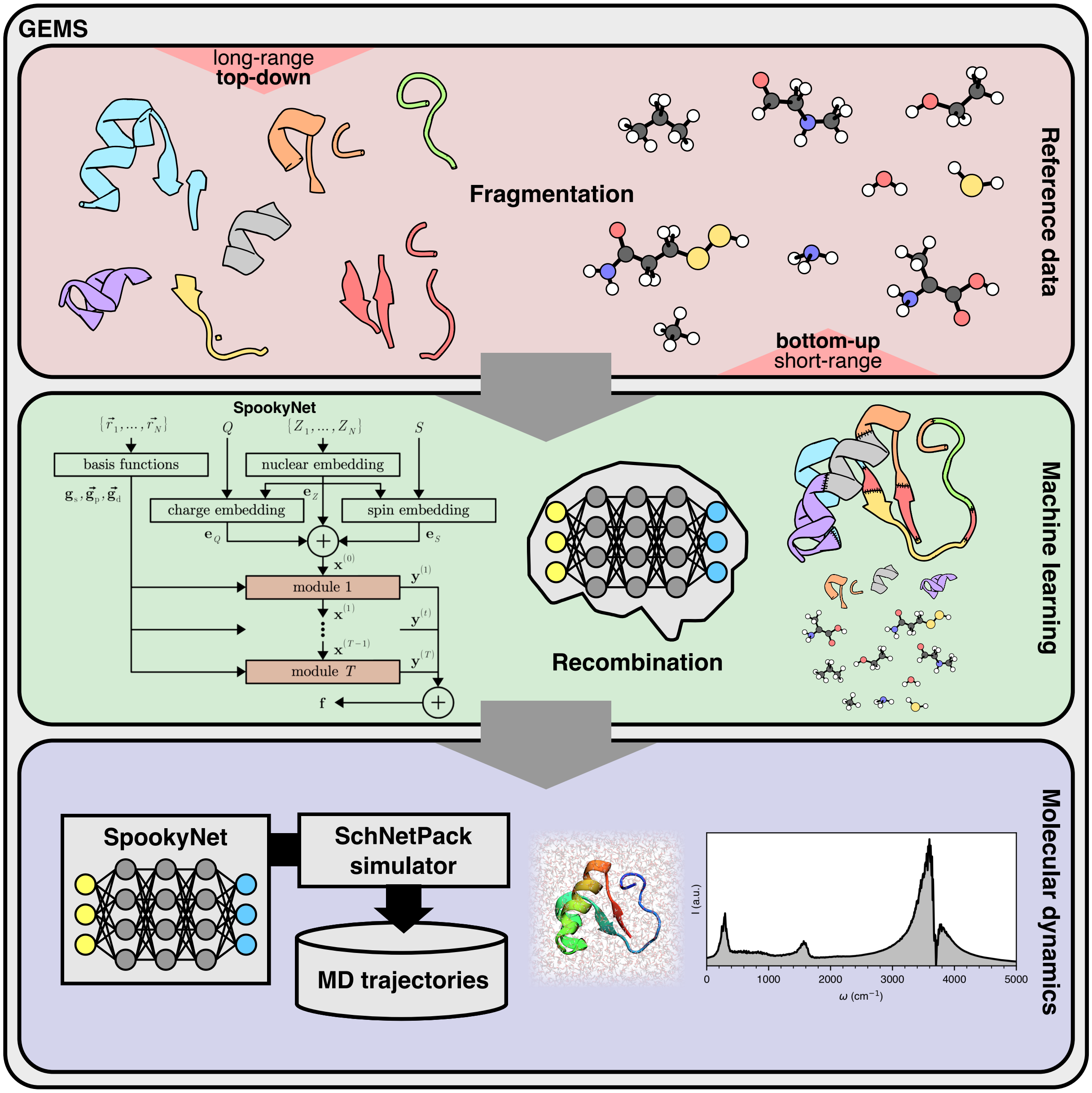

Figure S21: **GEMS method overview.** The GEMS method consists of three main steps: 1) Generation of reference data, 2) Training a machine learning model, and 3) Running MD simulations. In this work, step 1) is achieved by calculating PBE0+MBD reference data for a combination of bottom-up and top-down fragments. For step 2), we train a SpookyNet model, and for step 3) we use the MD package included in SchNetPack. However, all individual steps can be replaced in future work, for example, different reference data could be used, or another ML method instead of SpookyNet could be used as MLFF.

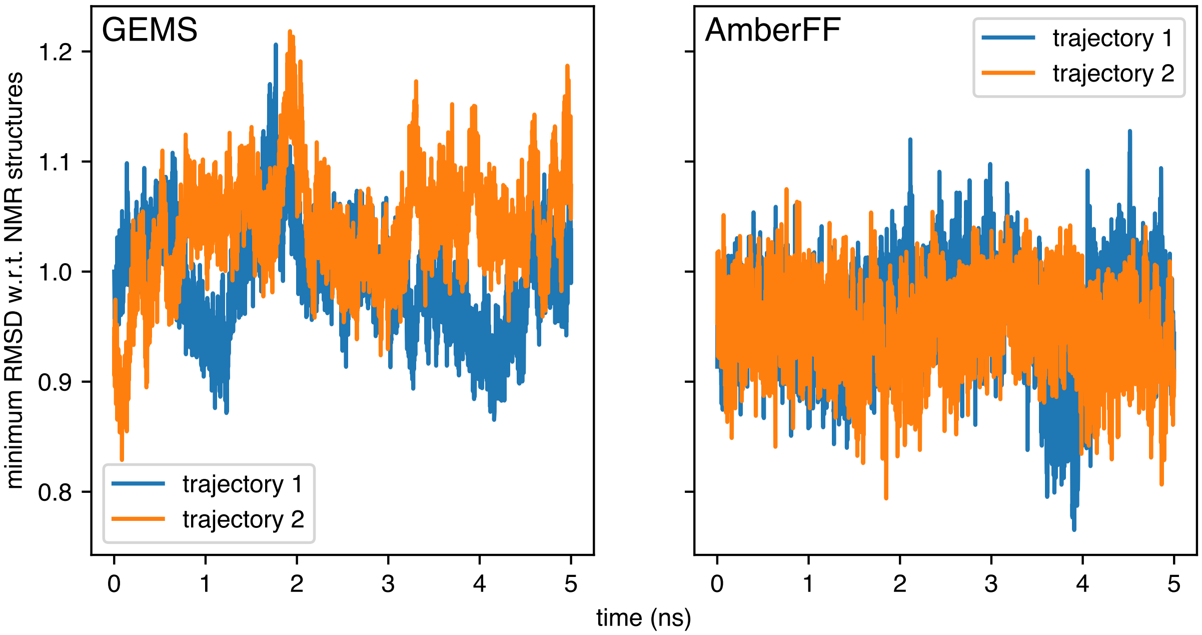

Figure S22: **RMSD (excluding hydrogen atoms) of crambin during GEMS and AmberFF trajectories with respect to 20 low energy water refined structures of crambin in dodecylphosphocholine micelles based on NMR measurements (*29*).** At each time point, the minimum RMSD to any of the 20 reference structures is shown. The average minimum RMSD of GEMS trajectories (1.00 Å and 1.04 Å) and AmberFF trajectories (0.96 Å and 0.95 Å) is comparable.

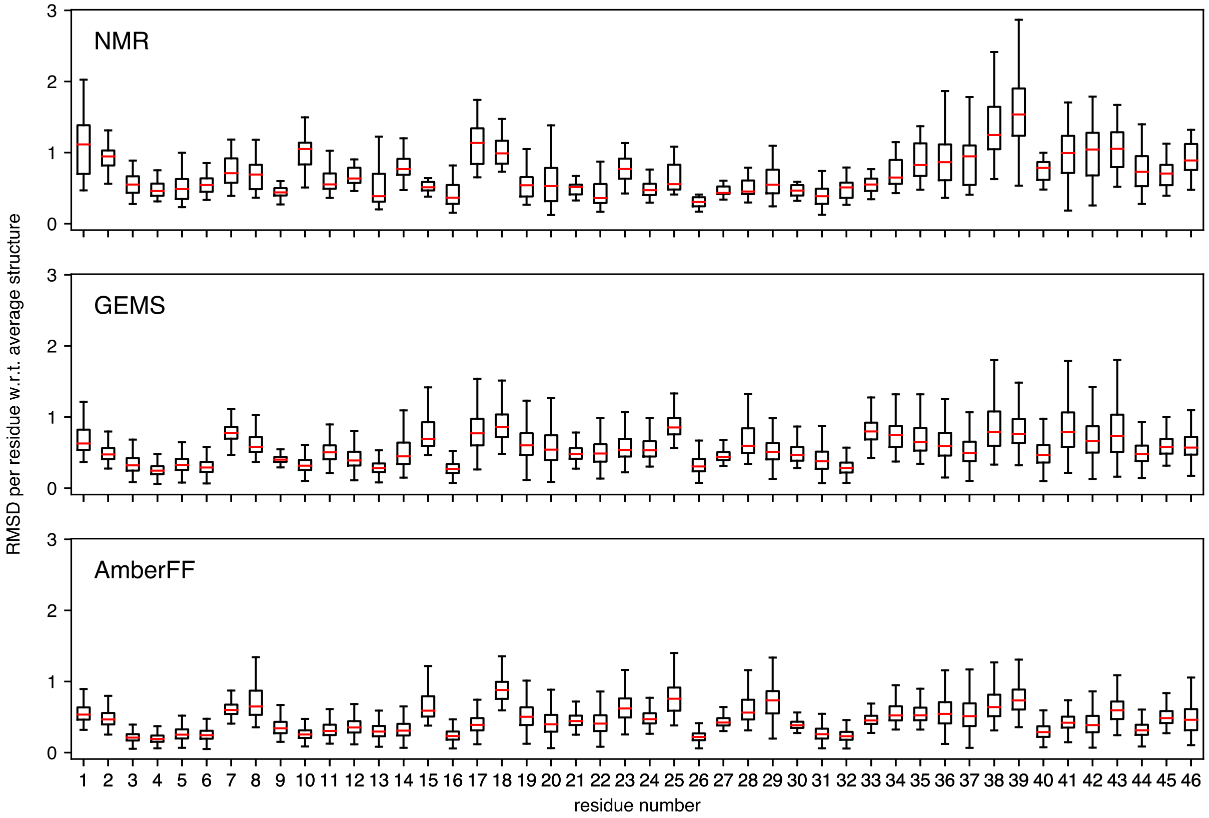

Figure S23: **RMSD per residue of crambin w.r.t. average structure.** A structural ensemble based on NMR measurements (*29*) (top) is compared to the structural ensemble sampled during MD simulations with GEMS (middle) and AmberFF (bottom). The box extends from the lower to upper quartile with whiskers extending to 1.5 times the interquartile range. A red line indicates the median value.

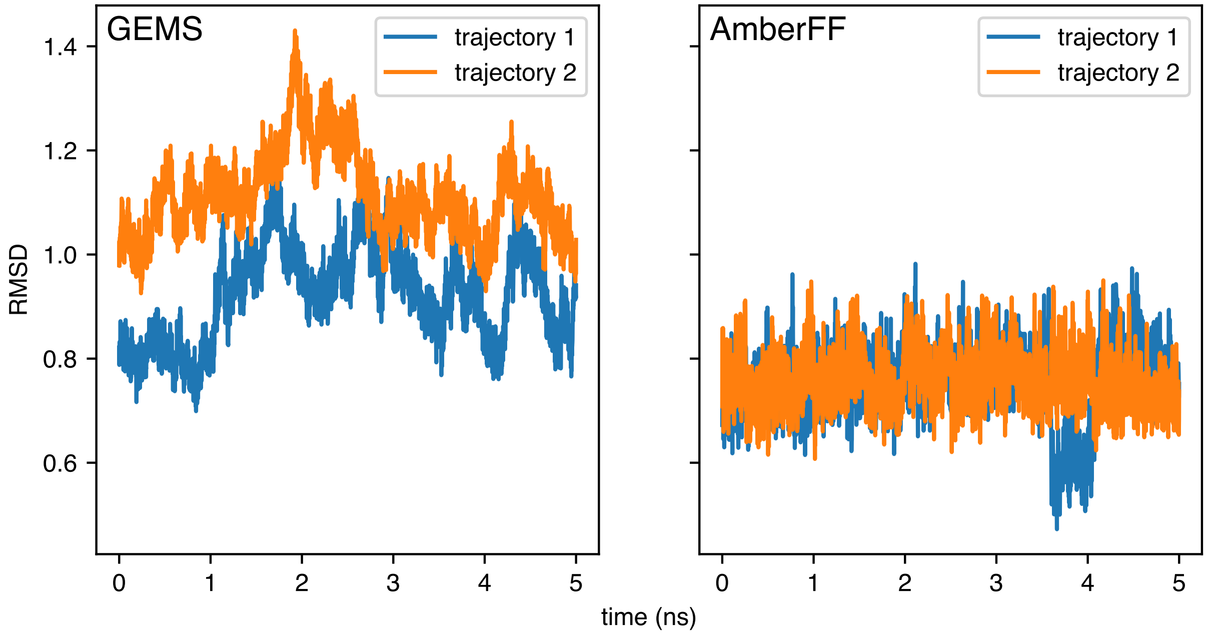

Figure S24: **RMSD (excluding hydrogen atoms) of crambin during GEMS and AmberFF trajectories with respect to a high-resolution crystal structure (PDB entry 1EJG (*75*)).** GEMS trajectories have a slightly larger average RMSD (0.92 Å and 1.12 Å) compared to AmberFF (0.75 Å and 0.76 Å), indicating that the structure is more flexible overall. Importantly, the RMSD of both GEMS and AmberFF trajectories does not increase over time, indicating that the folded structure of crambin is stable over the time scale of the simulation.

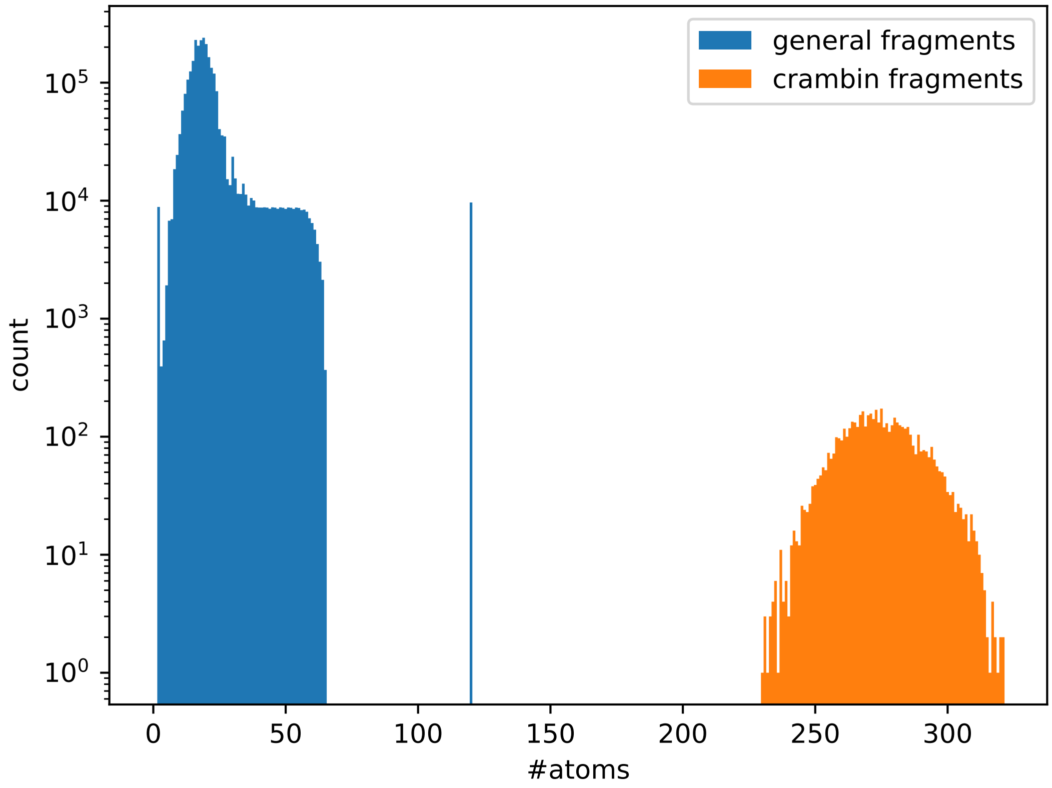

Figure S25: **Distribution of fragment size in the training data for training GEMS for crambin.** General fragments (blue) are not system-specific and relevant for all proteins in aqueous solution (the isolated peak at 120 atoms corresponds to structures consisting of 40 spherically arranged water molecules, resembling a “cutout” from bulk water). Crambin fragments (orange) were generated using the top-down approach described in the main text and are specific to crambin in solution.

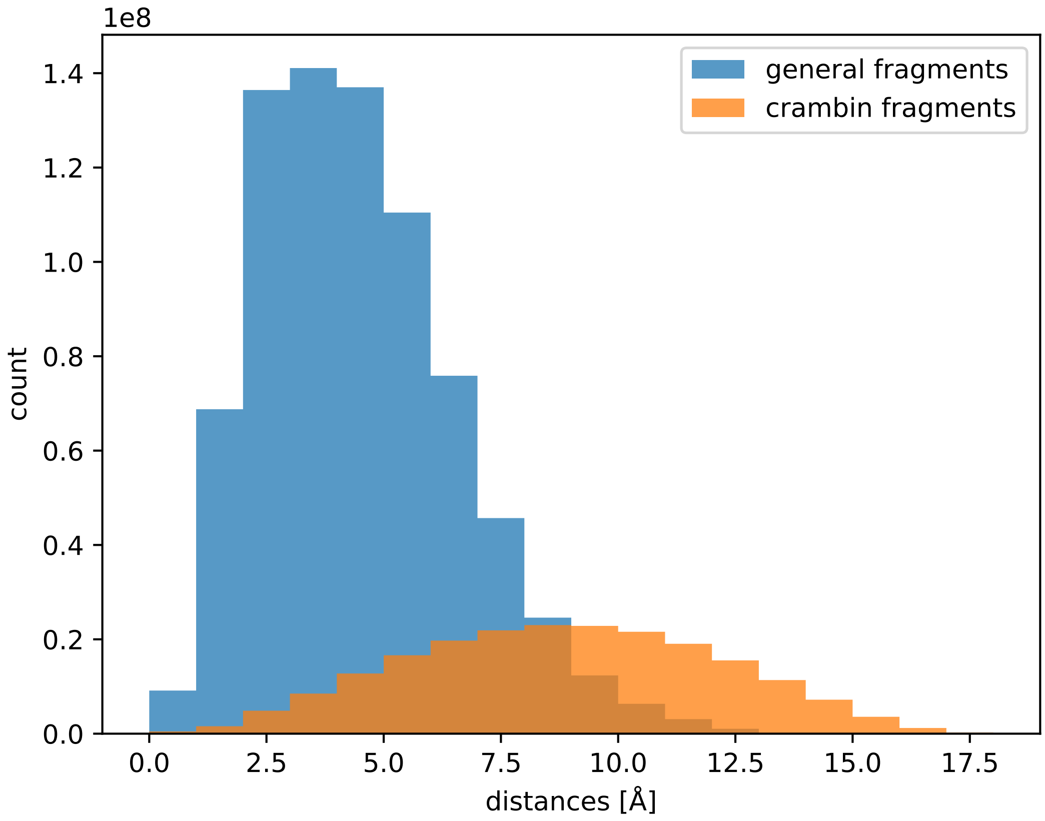

Figure S26: **Distribution of pairwise distances in the training data for training GEMS for crambin.** General fragments (blue) are not system-specific and relevant for all proteins in aqueous solution. Crambin fragments (orange) were generated using the top-down approach described in the main text and are specific to crambin in solution.

REFERENCES AND NOTES

1. M. Karplus, J. A. McCammon, Molecular dynamics simulations of biomolecules. *Nat. Struct. Biol.* **9**, 646–652 (2002).

2. F. Mouvet, J. Villard, V. Bolnykh, U. Röthlisberger, Recent advances in first-principles based molecular dynamics. *Acc. Chem. Res.* **55**, 221–230 (2022).

3. R. H. French, V. A. Parsegian, R. Podgornik, R. F. Rajter, A. Jagota, J. Luo, D. Asthagiri, M. K. Chaudhury, Y.-M. Chiang, S. Granick, Long range interactions in nanoscale science. *Rev. Mod. Phys.* **82**, 1887–1944 (2010).

4. D. E. Shaw, M. M. Deneroff, R. O. Dror, J. S. Kuskin, R. H. Larson, J. K. Salmon, C. Young, B. Batson, K. J. Bowers, J. C. Chao, M. P. Eastwood, J. Gagliardo, J. P. Grossman, C. R. Ho, D. J. Ierardi, I. Kolossváry, J. L. Klepeis, T. Layman, C. McLeavey, M. A. Moraes, R. Mueller, E. C. Priest, Y. Shan, J. Spengler, M. Theobald, B. Towles, S. C. Wang, Anton, a special-purpose machine for molecular dynamics simulation. *Commun. ACM* **51**, 91–97 (2008).

5. H. M. Senn, W. Thiel, QM/MM methods for biomolecular systems. *Angew. Chem. Int.* **48**, 1198–1229 (2009).

6. H. J. Kulik, J. Zhang, J. P. Klinman, T. J. Martinez, How large should the QM region be in QM/MM calculations? The case of catechol O-methyltransferase. *J. Phys. Chem. B* **120**, 11381–11394 (2016).

7. O. T. Unke, S. Chmiela, H. E. Sauceda, M. Gastegger, I. Poltavsky, K. T. Schütt, A. Tkatchenko, K.-R. Müller, Machine learning force fields. *Chem. Rev.* **121**, 10142–10186 (2021).

8. U. Rivero, O. T. Unke, M. Meuwly, S. Willitsch, Reactive atomistic simulations of Diels-Alder reactions: The importance of molecular rotations. *J. Chem. Phys.* **151**, 104301 (2019).

9. H. E. Sauceda, S. Chmiela, I. Poltavsky, K.-R. Müller, A. Tkatchenko, Molecular force fields with gradient-domain machine learning: Construction and application to dynamics of small molecules with coupled cluster forces. *J. Chem. Phys.* **150**, 114102 (2019).

10. S. Chmiela, V. Vassilev-Galindo, O. T. Unke, A. Kabylda, H. E. Sauceda, A. Tkatchenko, K.-R. Müller, Accurate global machine learning force fields for molecules with hundreds of atoms. *Sci. Adv.* **9**, eadf0873 (2023).

11. W. Jia, H. Wang, M. Chen, D. Lu, L. Lin, R. Car, E. Weinan, L. Zhang, *SC20: International Conference for High Performance Computing, Networking, Storage and Analysis* (IEEE, 2020), pp. 1–14.

12. M. Rossi, W. Fang, A. Michaelides, Stability of complex biomolecular structures: Van der Waals, hydrogen bond cooperativity, and nuclear quantum effects. *J. Phys. Chem. Lett.* **6**, 4233–4238 (2015).

13. M. Stöhr, A. Tkatchenko, Quantum mechanics of proteins in explicit water: The role of plasmon-like solute-solvent interactions. *Sci. Adv.* **5**, eaax0024 (2019).

14. Z. Cheng, J. Du, L. Zhang, J. Ma, W. Li, S. Li, Building quantum mechanics quality force fields of proteins with the generalized energy-based fragmentation approach and machine learning. *Phys. Chem. Chem. Phys.* **24**, 1326–1337 (2022).

15. Y. Han, Z. Wang, A. Chen, I. Ali, J. Cai, S. Ye, J. Li, An inductive transfer learning force field (ITLFF) protocol builds protein force fields in seconds. *Brief. Bioinform.* **23**, bbab590 (2022).

16. B. Huang, O. A. von Lilienfeld, Quantum machine learning using atom-in-molecule-based fragments selected on the fly. *Nat. Chem.* **12**, 945–951 (2020).

17. O. T. Unke, S. Chmiela, M. Gastegger, K. T. Schütt, H. E. Sauceda, K.-R. Müller, SpookyNet: Learning force fields with electronic degrees of freedom and nonlocal effects. *Nat. Commun.* **12**, 7273 (2021).

18. J. Behler, Representing potential energy surfaces by high-dimensional neural network potentials. *J. Condens. Matter Phys.* **26**, 183001 (2014).

19. O. T. Unke, M. Meuwly, PhysNet: A neural network for predicting energies, forces, dipole moments, and partial charges. *J. Chem. Theory Comput.* **15**, 3678–3693 (2019).

20. A. Grisafi, M. Ceriotti, Incorporating long-range physics in atomic-scale machine learning. *J. Chem. Phys.* **151**, 204105 (2019).

21. L. Zhang, H. Wang, M. C. Muniz, A. Z. Panagiotopoulos, R. Car, W. E , A deep potential model with long-range electrostatic interactions. *J. Chem. Phys.* **156**, 124107 (2022).

22. T. W. Ko, J. A. Finkler, S. Goedecker, J. Behler, A fourth-generation high-dimensional neural network potential with accurate electrostatics including non-local charge transfer. *Nat. Commun.* **12**, 1–11 (2021).

23. E. Balog, J. C. Smith, D. Perahia, Conformational heterogeneity and low-frequency vibrational modes of proteins. *Phys. Chem. Chem. Phys.* **8**, 5543–5548 (2006).

24. K. Lindorff-Larsen, S. Piana, K. Palmo, P. Maragakis, J. L. Klepeis, R. O. Dror, D. E. Shaw, Improved side-chain torsion potentials for the Amber ff99SB protein force field. *Proteins* **78**, 1950–1958 (2010).

25. M. Karplus, T. Ichiye, B. Pettitt, Configurational entropy of native proteins. *Biophys. J.* **52**, 1083–1085 (1987).

26. S. Stocker, J. Gasteiger, F. Becker, S. Günnemann, J. Margraf, How robust are modern graph neural network potentials in long and hot molecular dynamics simulations? *Mach. Learn. Sci. Technol.* **3**, 045010 (2022).

27. M. Gastegger, J. Behler, P. Marquetand, Machine learning molecular dynamics for the simulation of infrared spectra. *Chem. Sci.* **8**, 6924–6935 (2017).

28. C. Adamo, V. Barone, Toward reliable density functional methods without adjustable parameters: The PBE0 model. *J. Chem. Phys.* **110**, 6158–6170 (1999).

29. A. Tkatchenko, R. A. DiStasio Jr, R. Car, M. Scheffler, Accurate and efficient method for many-body van der Waals interactions. *Phys. Rev. Lett.* **108**, 236402 (2012).

30. F. Schubert, M. Rossi, C. Baldauf, K. Pagel, S. Warnke, G. von Helden, F. Filsinger, P. Kupser, G. Meijer, M. Salwiczek, B. Koksch, M. Scheffler, V. Blum, Exploring the conformational preferences of 20-residue peptides in isolation: Ac-Ala_{19}-Lys + H^+ vs. Ac-Lys-Ala_{19} + H^+ and the current reach of DFT. *Phys. Chem. Chem. Phys.* **17**, 7373–7385 (2015).

31. C. Baldauf, M. Rossi, Going clean: Structure and dynamics of peptides in the gas phase and paths to solvation. *J. Phys. Condens. Matter* **27**, 493002 (2015).

32. J. Hermann, D. Alfè, A. Tkatchenko, Nanoscale π- π stacked molecules are bound by collective charge fluctuations. *Nat. Commun.* **8**, 14052 (2017).

33. J. Hoja, H.-Y. Ko, M. A. Neumann, R. Car, R. A. DiStasio, A. Tkatchenko, Reliable and practical computational description of molecular crystal polymorphs. *Sci. Adv.* **5**, eaau3338 (2019).

34. D. Firaha, Y. M. Liu, J. van de Streek, K. Sasikumar, H. Dietrich, J. Helfferich, L. Aerts, D. E. Braun, A. Broo, A. G. DiPasquale, A. Y. Lee, S. le Meur, S. O. Nilsson Lill, W. J. Lunsmann, A. Mattei, P. Muglia, O. D. Putra, M. Raoui, S. M. Reutzel-Edens, S. Rome, A. Y. Sheikh, A. Tkatchenko, G. R. Woollam, M. A. Neumann, Predicting crystal form stability under real-world conditions. *Nature* **623**, 324–328 (2023).

35. R. A. DiStasio Jr, B. Santra, Z. Li, X. Wu, R. Car, The individual and collective effects of exact exchange and dispersion interactions on the ab initio structure of liquid water. *J. Chem. Phys.* **141**, 084502 (2014).

36. C. Park, W. A. Goddard, Stabilization of α-helices by dipole−dipole interactions within α-helices. *J. Phys. Chem. B* **104**, 7784–7789 (2000).

37. M. Kohtani, T. C. Jones, J. E. Schneider, M. F. Jarrold, Extreme stability of an unsolvated α-helix. *J. Am. Chem. Soc.* **126**, 7420–7421 (2004).

38. A. Tkatchenko, M. Rossi, V. Blum, J. Ireta, M. Scheffler, Unraveling the stability of polypeptide helices: Critical role of van der Waals interactions. *Phys. Rev. Lett.* **106**, 118102 (2011).

39. I. A. Topol, S. K. Burt, E. Deretey, T.-H. Tang, A. Perczel, A. Rashin, I. G. Csizmadia, α- and 3_{10}-Helix Interconversion: A quantum-chemical study on polyalanine systems in the gas phase and in aqueous solvent. *J. Am. Chem. Soc.* **123**, 6054–6060 (2001).

40. G. L. Millhauser, C. J. Stenland, P. Hanson, K. A. Bolin, F. J. van de Ven, Estimating the relative populations of 3_{10}-helix and α-helix in Ala-rich peptides: A hydrogen exchange and high field NMR study. *J. Mol. Biol.* **267**, 963–974 (1997).

41. K. A. Bolin, G. L. Millhauser, α and 3_{10}: The split personality of polypeptide helices. *Acc. Chem. Res.* **32**, 1027–1033 (1999).

42. N. Foloppe, A. D. MacKerell, Jr, All-atom empirical force field for nucleic acids: I. parameter optimization based on small molecule and condensed phase macromolecular target data. *J. Comput. Chem.* **21**, 86–104 (2000).

43. C. Oostenbrink, A. Villa, A. E. Mark, W. F. Van Gunsteren, A biomolecular force field based on the free enthalpy of hydration and solvation: The GROMOS force-field parameter sets 53A5 and 53A6. *J. Comput. Chem.* **25**, 1656–1676 (2004).

44. F. Weigend, R. Ahlrichs, Balanced basis sets of split valence, triple zeta valence and quadruple zeta valence quality for H to Rn: Design and assessment of accuracy. *Phys. Chem. Chem. Phys.* **7**, 3297–3305 (2005).

45. M. Thomas, M. Brehm, R. Fligg, P. Vöhringer, B. Kirchner, Computing vibrational spectra from ab initio molecular dynamics. *Phys. Chem. Chem. Phys.* **15**, 6608–6622 (2013).

46. C. D. Craver, *The Coblentz Society Desk Book of Infrared Spectra* (National Standard Reference Data System, 1977).

47. H.-C. Ahn, N. Juranić, S. Macura, J. L. Markley, Three-dimensional structure of the water-insoluble protein crambin in dodecylphosphocholine micelles and its minimal solvent-exposed surface. *J. Am. Chem. Soc.* **128**, 4398–4404 (2006).

48. K. N. Woods, The glassy state of crambin and the THz time scale protein-solvent fluctuations possibly related to protein function. *BMC Biophys.* **7**, 1–15 (2014).

49. D. A. Case, Molecular dynamics and NMR spin relaxation in proteins. *Acc. Chem. Res.* **35**, 325–331 (2002).

50. S. Piana, K. Lindorff-Larsen, D. E. Shaw, How robust are protein folding simulations with respect to force field parameterization? *Biophys. J.* **100**, L47–L49 (2011).

51. R. M. Levy, D. Perahia, M. Karplus, Molecular dynamics of an α-helical polypeptide: Temperature dependence and deviation from harmonic behavior. *Proc. Natl. Acad. Sci. U.S.A.* **79**, 1346–1350 (1982).

52. J. A. McCammon, B. R. Gelin, M. Karplus, Dynamics of folded proteins. *Nature* **267**, 585–590 (1977).

53. D. Phillips, *Biomolecular Stereodynamics* (Adenine Press, 1981).

54. H. E. Sauceda, V. Vassilev-Galindo, S. Chmiela, K.-R. Müller, A. Tkatchenko, Dynamical strengthening of covalent and non-covalent molecular interactions by nuclear quantum effects at finite temperature. *Nat. Commun.* **12**, 442 (2021).

55. B. Hess, C. Kutzner, D. Van Der Spoel, E. Lindahl, GROMACS 4: Algorithms for highly efficient, load-balanced, and scalable molecular simulation. *J. Chem. Theory Comput.* **4**, 435–447 (2008).

56. K. Saravanan, J. R. Kitchin, O. A. Von Lilienfeld, J. A. Keith, Alchemical predictions for computational catalysis: Potential and limitations. *J. Phys. Chem. Lett.* **8**, 5002–5007 (2017).

57. F. R. Beierlein, G. G. Kneale, T. Clark, Predicting the effects of basepair mutations in DNA-protein complexes by thermodynamic integration. *Biophys. J.* **101**, 1130–1138 (2011).

58. M. Krepl, M. Otyepka, P. Banáš, J. Šponer, Effect of guanine to inosine substitution on stability of canonical DNA and RNA duplexes: Molecular dynamics thermodynamics integration study. *J. Phys. Chem. B* **117**, 1872–1879 (2013).

59. E. Di Cera, Site-specific thermodynamics: Understanding cooperativity in molecular recognition. *Chem. Rev.* **98**, 1563–1592 (1998).

60. A. S. Raman, K. I. White, R. Ranganathan, Origins of allostery and evolvability in proteins: A case study. *Cell* **166**, 468–480 (2016).

61. D. Wrapp, N. Wang, K. S. Corbett, J. A. Goldsmith, C.-L. Hsieh, O. Abiona, B. S. Graham, J. S. McLellan, Cryo-EM structure of the 2019-nCoV spike in the prefusion conformation. *Science* **367**, 1260–1263 (2020).

62. N. M. O'Boyle, M. Banck, C. A. James, C. Morley, T. Vandermeersch, G. R. Hutchison, Open Babel: An open chemical toolbox. *J. Chem.* **3**, 1–14 (2011).

63. O. Unke, M. Meuwly, Solvated protein fragments data set (2019); https://doi.org/10.5281/zenodo.2605371.

64. R. M. Parrish, L. A. Burns, D. G. A. Smith, A. C. Simmonett, A. E. DePrince, E. G. Hohenstein, U. Bozkaya, A. Y. Sokolov, R. Di Remigio, R. M. Richard, J. F. Gonthier, A. M. James, H. R. McAlexander, A. Kumar, M. Saitow, X. Wang, B. P. Pritchard, P. Verma, H. F. Schaefer, K. Patkowski, R. A. King, E. F. Valeev, F. A. Evangelista, J. M. Turney, T. D. Crawford, C. D. Sherrill, Psi4 1.1: An open-source electronic structure program emphasizing automation, advanced libraries, and interoperability. *J. Chem. Theory Comput.* **13**, 3185–3197 (2017).

65. J. T. Barron, A general and adaptive robust loss function. *Proc. IEEE Int. Conf. Comput. Vis.*, 4331–4339 (2019).

66. L. Konermann, H. Metwally, R. G. McAllister, V. Popa, How to run molecular dynamics simulations on electrospray droplets and gas phase proteins: Basic guidelines and selected applications. *Methods* **144**, 104–112 (2018).

67. G. Bussi, D. Donadio, M. Parrinello, Canonical sampling through velocity rescaling. *J. Chem. Phys.* **126**, 014101 (2007).

68. M. Parrinello, A. Rahman, Polymorphic transitions in single crystals: A new molecular dynamics method. *J. Appl. Phys.* **52**, 7182–7190 (1981).

69. M. D. Hanwell, D. E. Curtis, D. C. Lonie, T. Vandermeersch, E. Zurek, G. R. Hutchison, Avogadro: An advanced semantic chemical editor, visualization, and analysis platform. *J. Chem.* **4**, 1–17 (2012).

70. D. Bang, V. Tereshko, A. A. Kossiakoff, S. B. Kent, Role of a salt bridge in the model protein crambin explored by chemical protein synthesis: X-ray structure of a unique protein analogue, [V15A]crambin-α-carboxamide. *Mol. Biosyst.* **5**, 750–756 (2009).

71. W. L. DeLano, PyMOL: An open-source molecular graphics tool. *CCP4 Newsl. Protein Crystallogr.* **40**, 82–92 (2002).

72. C. Jelsch, M. M. Teeter, V. Lamzin, V. Pichon-Pesme, R. H. Blessing, C. Lecomte, Accurate protein crystallography at ultra-high resolution: Valence electron distribution in crambin. *Proc. Natl. Acad. Sci. U.S.A.* **97**, 3171–3176 (2000).

73. A. Schmidt, M. Teeter, E. Weckert, V. S. Lamzin, Crystal structure of small protein crambin at 0.48 Å resolution. *Acta Crystallogr. F* **67**, 424–428 (2011).

74. J. M. Delgado, N. Duro, D. M. Rogers, A. Tkatchenko, S. A. Pandit, S. Varma, Molecular basis for higher affinity ofSARS-CoV-2 spikeRBDfor humanACE2receptor. *Proteins* **89**, 1134–1144 (2021).

75. K. Schütt, P. Kessel, M. Gastegger, K. Nicoli, A. Tkatchenko, K.-R. Müller, SchNetPack: A deep learning toolbox for atomistic systems. *J. Chem. Theory Comput.* **15**, 448–455 (2018).

76. G. J. Martyna, M. E. Tuckerman, D. J. Tobias, M. L. Klein, Explicit reversible integrators for extended systems dynamics. *Mol. Phys.* **87**, 1117–1157 (1996).

77. L. Wilson, R. Krasny, T. Luchko, Accelerating the 3d reference interaction site model theory of molecular solvation with treecode summation and cut-offs. *J. Comput. Chem.* **43**, 1251–1270 (2022).

78. G. Bussi, M. Parrinello, Accurate sampling using Langevin dynamics. *Phys. Rev. B* **75**, 056707 (2007).

79. D. Frishman, P. Argos, Knowledge-based protein secondary structure assignment. *Proteins* **23**, 566–579 (1995).

80. L. McInnes, J. Healy, J. Melville, UMAP: Uniform manifold approximation and projection for dimension reduction. arXiv:1802.03426 [stat.ML] (2018).

81. P. L. Freddolino, A. S. Arkhipov, S. B. Larson, A. McPherson, K. Schulten, Molecular dynamics simulations of the complete satellite tobacco mosaic virus. *Structure* **14**, 437–449 (2006).

82. G. Zhao, J. R. Perilla, E. L. Yufenyuy, X. Meng, B. Chen, J. Ning, J. Ahn, A. M. Gronenborn, K. Schulten, C. Aiken, P. Zhang , Mature HIV-1 capsid structure by cryo-electron microscopy and all-atom molecular dynamics. *Nature* **497**, 643–646 (2013).

83. M. I. Zimmerman, J. R. Porter, M. D. Ward, S. Singh, N. Vithani, A. Meller, U. L. Mallimadugula, C. E. Kuhn, J. H. Borowsky, R. P. Wiewiora, Citizen scientists create an exascale computer to combat COVID-19. bioRxiv 2020.06.27.175430 [Preprint] (2020). https://doi.org/10.1101/2020.06.27.175430.

84. J. E. Lennard-Jones, On the determination of molecular fields—II. From the equation of state of a gas. *Proc. R. Soc. Lond. A* **106**, 463–477 (1924).

85. M. González, *École Thématique de la Société Française de la Neutronique* (EDP Sciences, 2011), vol. **12**, pp. 169–200.

86. O. T. Unke, D. Koner, S. Patra, S. Käser, M. Meuwly, High-dimensional potential energy surfaces for molecular simulations: From empiricism to machine learning. *Mach. Learn. Sci. Technol.* **1**, 13001 (2020).

87. F. Vitalini, A. S. Mey, F. Noé, B. G. Keller, Dynamic properties of force fields. *J. Chem. Phys.* **142**, 02B611_1 (2015).

88. T. A. Halgren, W. Damm, Polarizable force fields. *Curr. Opin. Struct. Biol.* **11**, 236–242 (2001).

89. A. Warshel, M. Kato, A. V. Pisliakov, Polarizable force fields: History, test cases, and prospects. *J. Chem. Theory Comput.* **3**, 2034–2045 (2007).

90. T. D. Rasmussen, P. Ren, J. W. Ponder, F. Jensen, Force field modeling of conformational energies: Importance of multipole moments and intramolecular polarization. *Int. J. Quantum Chem.* **107**, 1390–1395 (2007).

91. M. G. Darley, C. M. Handley, P. L. Popelier, Beyond point charges: Dynamic polarization from neural net predicted multipole moments. *J. Chem. Theory Comput.* **4**, 1435–1448 (2008).

92. S. M. Kandathil, T. L. Fletcher, Y. Yuan, J. Knowles, P. L. Popelier, Accuracy and tractability of a Kriging model of intramolecular polarizable multipolar electrostatics and its application to histidine. *J. Comput. Chem.* **34**, 1850–1861 (2013).

93. S. Cardamone, T. J. Hughes, P. L. Popelier, Multipolar electrostatics. *Phys. Chem. Chem. Phys.* **16**, 10367–10387 (2014).

94. O. T. Unke, M. Devereux, M. Meuwly, Minimal distributed charges: Multipolar quality at the cost of point charge electrostatics. *J. Chem. Phys.* **147**, 161712 (2017).

95. A. Warshel, R. M. Weiss, An empirical valence bond approach for comparing reactions in solutions and in enzymes. *J. Am. Chem. Soc.* **102**, 6218–6226 (1980).

96. A. C. Van Duin, S. Dasgupta, F. Lorant, W. A. Goddard, ReaxFF: A reactive force field for hydrocarbons. *J. Phys. Chem. A* **105**, 9396–9409 (2001).

97. J. Behler, M. Parrinello, Generalized neural-network representation of high-dimensional potential-energy surfaces. *Phys. Rev. Lett.* **98**, 146401 (2007).

98. P. L. Popelier, QCTFF: On the construction of a novel protein force field. *Int. J. Quantum Chem.* **115**, 1005–1011 (2015).

99. J. S. Smith, O. Isayev, A. E. Roitberg, ANI-1: An extensible neural network potential with DFT accuracy at force field computational cost. *Chem. Sci.* **8**, 3192–3203 (2017).

100. J. Behler, First principles neural network potentials for reactive simulations of large molecular and condensed systems. *Angew. Chem. Int. Ed.* **56**, 12828–12840 (2017).

101. J. Behler, Four generations of high-dimensional neural network potentials. *Chem. Rev.* **121**, 10037–10072 (2021).

102. S. Chmiela, A. Tkatchenko, H. E. Sauceda, I. Poltavsky, K. T. Schütt, K.-R. Müller, Machine learning of accurate energy-conserving molecular force fields. *Sci. Adv.* **3**, e1603015 (2017).

103. S. Chmiela, H. E. Sauceda, K.-R. Müller, A. Tkatchenko, Towards exact molecular dynamics simulations with machine-learned force fields. *Nat. Commun.* **9**, 3887 (2018).

104. K. T. Schütt, S. Chmiela, O. A. von Lilienfeld, A. Tkatchenko, K. Tsuda, K.-R. Müller, Machine learning meets quantum physics, in *Lecture Notes in Physics* (Springer, 2020).

105. M. Rupp, A. Tkatchenko, K.-R. Müller, O. A. Von Lilienfeld, Fast and accurate modeling of molecular atomization energies with machine learning. *Phys. Rev. Lett.* **108**, 058301 (2012).

106. G. Montavon, M. Rupp, V. Gobre, A. Vazquez-Mayagoitia, K. Hansen, A. Tkatchenko, K.-R. Müller, O. A. Von Lilienfeld, Machine learning of molecular electronic properties in chemical compound space. *New J. Phys.* **15**, 095003 (2013).

107. K. Hansen, G. Montavon, F. Biegler, S. Fazli, M. Rupp, M. Scheffler, O. A. Von Lilienfeld, A. Tkatchenko, K.-R. Müller, Assessment and validation of machine learning methods for predicting molecular atomization energies. *J. Chem. Theory Comput.* **9**, 3404–3419 (2013).

108. K. Hansen, F. Biegler, R. Ramakrishnan, W. Pronobis, O. A. Von Lilienfeld, K.-R. Muller, A. Tkatchenko, Machine learning predictions of molecular properties: Accurate many-body potentials and nonlocality in chemical space. *J. Phys. Chem. Lett.* **6**, 2326–2331 (2015).

109. L. Boninsegna, F. Nüske, C. Clementi, Sparse learning of stochastic dynamical equations. *J. Chem. Phys.* **148**, 241723 (2018).

110. F. Noé, S. Olsson, J. Köhler, H. Wu, Boltzmann generators: Sampling equilibrium states of many-body systems with deep learning. *Science* **365**, eaaw1147 (2019).

111. J. Köhler, L. Klein, F. Noé, Equivariant flows: Exact likelihood generative learning for symmetric densities. arXiv:2006.02425 [stat.ML] (2020).

112. J. Zhang, Y. I. Yang, F. Noé, Targeted adversarial learning optimized sampling. *J. Phys. Chem. Lett.* **10**, 5791–5797 (2019).

113. D. Koner, O. T. Unke, K. Boe, R. J. Bemish, M. Meuwly, Exhaustive state-to-state cross sections for reactive molecular collisions from importance sampling simulation and a neural network representation. *J. Chem. Phys.* **150**, 211101 (2019).

114. A. W. Senior, R. Evans, J. Jumper, J. Kirkpatrick, L. Sifre, T. Green, C. Qin, Zidek A, A. W. Nelson, A. Bridgland, H. Penedones, S. Petersen, K. Simonyan, S. Crossan, P. Kohli, D. T. Jones, D. Silver, K. Kavukcuoglu, D. Hassabis, Improved protein structure prediction using potentials from deep learning. *Nature* **577**, 706–710 (2020).

115. J. Jumper, R. Evans, A. Pritzel, T. Green, M. Figurnov, O. Ronneberger, K. Tunyasuvunakool, R. Bates, Zidek A, A. Potapenko, A. Bridgland, C. Meyer, S. A. A. Kohl, A. J. Ballard, A. Cowie, B. Romera-Paredes, S. Nikolov, R. Jain, J. Adler, T. Back, S. Petersen, D. Reiman, E. Clancy, M. Zielinski, M. Steinegger, M. Pacholska, T. Berghammer, S. Bodenstein, D. Silver, O.

Vinyals, A. W. Senior, K. Kavukcuoglu, P. Kohli, D. Hassabis, Highly accurate protein structure prediction with AlphaFold. *Nature* ***596***, 583–589 (2021).

116. K. Tunyasuvunakool, J. Adler, Z. Wu, T. Green, M. Zielinski, Zidek A, A. Bridgland, A. Cowie, C. Meyer, A. Laydon, S. Velankar, G. J. Kleywegt, A. Bateman, R. Evans, A. Pritzel, M. Figurnov, O. Ronneberger, R. Bates, S. A. A. Kohl, A. Potapenko, A. J. Ballard, B. Romera-Paredes, S. Nikolov, R. Jain, E. Clancy, D. Reimann, S. Petersen, A. W. Senior, K. Kavukcuoglu, E. Birney, P. Kohli, J. Jumper, D. Hassabis, Highly accurate protein structure prediction for the human proteome. *Nature* **596**, 590–596 (2021).

117. G. Carleo, M. Troyer, Solving the quantum many-body problem with artificial neural networks. *Science* **355**, 602–606 (2017).

118. D. Pfau, J. S. Spencer, A. G. Matthews, W. M. C. Foulkes, *Ab* initiosolution of the many-electron Schrödinger equation with deep neural networks. *Phys. Rev. Res.* **2**, 033429 (2020).

119. J. Hermann, Z. Schätzle, F. Noé, Deep-neural-network solution of the electronic Schrödinger equation. *Nat. Chem.* **12**, 891–897 (2020).

120. K. Schütt, M. Gastegger, A. Tkatchenko, K.-R. Müller, R. J. Maurer, Unifying machine learning and quantum chemistry with a deep neural network for molecular wavefunctions. *Nat. Commun.* **10**, 5024 (2019).

121. M. Gastegger, A. McSloy, M. Luya, K. Schütt, R. Maurer, A deep neural network for molecular wave functions in quasi-atomic minimal basis representation. *J. Chem. Phys.* **153**, 044123 (2020).

122. O. Unke, M. Bogojeski, M. Gastegger, M. Geiger, T. Smidt, K.-R. Müller, SE(3)-equivariant prediction of molecular wavefunctions and electronic densities. *Adv. Neur. Inform. Process. Syst.* **34**, 1 (2021).

123. M. Gastegger, K. T. Schütt, K.-R. Müller, Machine learning of solvent effects on molecular spectra and reactions. *Chem. Sci.* **12**, 11473–11483 (2021).

124. M. Popova, O. Isayev, A. Tropsha, Deep reinforcement learning for *de novo* drug design. *Sci. Adv.* **4**, eaap7885 (2018).

125. M. Popova, M. Shvets, J. Oliva, O. Isayev, MolecularRNN: Generating realistic molecular graphs with optimized properties. arXiv:1905.13372 [cs.LG] (2019).

126. N. W. Gebauer, M. Gastegger, K. T. Schütt, *NeurIPS 2018 Workshop on Machine Learning for Molecules and Materials* (2018).

127. N. Gebauer, M. Gastegger, K. Schütt, Symmetry-adapted generation of 3d point sets for the targeted discovery of molecules. *Adv. Neural. Inf. Process. Syst.*, 7566–7578 (2019).

128. M. Hoffmann, F. Noé, Generating valid Euclidean distance matrices. arXiv:1910.03131 [cs.LG] (2019).

129. R. Winter, F. Montanari, A. Steffen, H. Briem, F. Noé, D.-A. Clevert, Efficient multi-objective molecular optimization in a continuous latent space. *Chem. Sci.* **10**, 8016–8024 (2019).

130. N. W. Gebauer, M. Gastegger, S. S. Hessmann, K.-R. Müller, K. T. Schütt, Inverse design of 3d molecular structures with conditional generative neural networks. *Nat. Commun.* **13**, 973 (2022).

131. F. Strieth-Kalthoff, F. Sandfort, M. H. Segler, F. Glorius, Machine learning the ropes: Principles, applications and directions in synthetic chemistry. *Chem. Soc. Rev.* **49**, 6154–6168 (2020).

132. A. Jain, S. P. Ong, G. Hautier, W. Chen, W. D. Richards, S. Dacek, S. Cholia, D. Gunter, D. Skinner, G. Ceder, K. A. Persson , Commentary: The materials project: A materials genome approach to accelerating materials innovation. *APL Mater.* **1**, 011002 (2013).

133. C. C. Fischer, K. J. Tibbetts, D. Morgan, G. Ceder, Predicting crystal structure by merging data mining with quantum mechanics. *Nat. Mater.* **5**, 641–646 (2006).

134. D. P. Tabor, L. M. Roch, S. K. Saikin, C. Kreisbeck, D. Sheberla, J. H. Montoya, S. Dwaraknath, M. Aykol, C. Ortiz, H. Tribukait, C. Amador-Bedolla, C. J. Brabec, B. Maruyama, K. A. Persson, A. Aspuru-Guzik , Accelerating the discovery of materials for clean energy in the era of smart automation. *Nat. Rev. Mater.* **3**, 5–20 (2018).

135. K. T. Butler, D. W. Davies, H. Cartwright, O. Isayev, A. Walsh, Machine learning for molecular and materials science. *Nature* **559**, 547–555 (2018).

136. P. De Luna, J. Wei, Y. Bengio, A. Aspuru-Guzik, E. Sargent, Use machine learning to find energy materials. *Nature* **552**, 23–27 (2017).

137. G. L. Hart, T. Mueller, C. Toher, S. Curtarolo, Machine learning for alloys. *Nat. Rev. Mater.* **6**, 730–755 (2021).

138. F. Noé, A. Tkatchenko, K.-R. Müller, C. Clementi, Machine learning for molecular simulation. *Annu. Rev. Phys. Chem.* **71**, 361–390 (2020).

139. O. A. von Lilienfeld, K.-R. Müller, A. Tkatchenko, Exploring chemical compound space with quantum-based machine learning. *Nat. Rev. Chem.* **4**, 347–358 (2020).

140. B. Huang, O. A. von Lilienfeld, *Ab initio* machine learning in chemical compound space. *Chem. Rev.* **121**, 10001–10036 (2021).

141. O. A. von Lilienfeld, K. Burke, Retrospective on a decade of machine learning for chemical discovery. *Nat. Commun.* **11**, 4895 (2020).

142. A. Tkatchenko, Machine learning for chemical discovery. *Nat. Commun.* **11**, 4125 (2020).

143. J. A. Keith, V. Vassilev-Galindo, B. Cheng, S. Chmiela, M. Gastegger, K.-R. Müller, A. Tkatchenko, Combining machine learning and computational chemistry for predictive insights into chemical systems. *Chem. Rev.* **121**, 9816–9872 (2021).

144. A. Glielmo, B. E. Husic, A. Rodriguez, C. Clementi, F. Noé, A. Laio, Unsupervised learning methods for molecular simulation data. *Chem. Rev.* **121**, 9722–9758 (2021).

145. M. Meuwly, Machine learning for chemical reactions. *Chem. Rev.* **121**, 10218–10239 (2021).

146. J. Westermayr, P. Marquetand, Machine learning for electronically excited states of molecules. *Chem. Rev.* **121**, 9873–9926 (2021).

147. B. J. Frey, D. Dueck, Clustering by passing messages between data points. *Science* **315**, 972–976 (2007).

148. A. H. Larsen, J. J. Mortensen, J. Blomqvist, I. E. Castelli, R. Christensen, M. Dułak, J. Friis, M. N. Groves, B. Hammer, C. Hargus, The atomic simulation environment—A Python library for working with atoms. *J. Phys. Condens. Matter* **29**, 273002 (2017).

149. J. A. Morrone, R. Car, Nuclear quantum effects in water. *Phys. Rev. Lett.* **101**, 017801 (2008).

150. G. Hura, J. M. Sorenson, R. M. Glaeser, T. Head-Gordon, A high-quality x-ray scattering experiment on liquid water at ambient conditions. *J. Chem. Phys.* **113**, 9140–9148 (2000).
